# Genomic prediction applied to high-biomass sorghum for bioenergy production

**DOI:** 10.1007/s11032-018-0802-5

**Published:** 2018-04-10

**Authors:** Amanda Avelar de Oliveira, Maria Marta Pastina, Vander Filipe de Souza, Rafael Augusto da Costa Parrella, Roberto Willians Noda, Maria Lúcia Ferreira Simeone, Robert Eugene Schaffert, Jurandir Vieira de Magalhães, Cynthia Maria Borges Damasceno, Gabriel Rodrigues Alves Margarido

**Affiliations:** 1 Department of Genetics, Luiz de Queiroz College of Agriculture, University of São Paulo, Piracicaba, SP 13418-900 Brazil; 2 Embrapa Maize and Sorghum, Sete Lagoas, Minas Gerais 35701-970 Brazil

**Keywords:** Bioenergy, Predictive models, Accuracy, Genotyping by sequencing, Functional enrichment

## Abstract

The increasing cost of energy and finite oil and gas reserves have created a need to develop alternative fuels from renewable sources. Due to its abiotic stress tolerance and annual cultivation, high-biomass sorghum (*Sorghum bicolor* L. Moench) shows potential as a bioenergy crop. Genomic selection is a useful tool for accelerating genetic gains and could restructure plant breeding programs by enabling early selection and reducing breeding cycle duration. This work aimed at predicting breeding values via genomic selection models for 200 sorghum genotypes comprising landrace accessions and breeding lines from biomass and saccharine groups. These genotypes were divided into two sub-panels, according to breeding purpose. We evaluated the following phenotypic biomass traits: days to flowering, plant height, fresh and dry matter yield, and fiber, cellulose, hemicellulose, and lignin proportions. Genotyping by sequencing yielded more than 258,000 single-nucleotide polymorphism markers, which revealed population structure between subpanels. We then fitted and compared genomic selection models BayesA, BayesB, BayesCπ, BayesLasso, Bayes Ridge Regression and random regression best linear unbiased predictor. The resulting predictive abilities varied little between the different models, but substantially between traits. Different scenarios of prediction showed the potential of using genomic selection results between sub-panels and years, although the genotype by environment interaction negatively affected accuracies. Functional enrichment analyses performed with the marker-predicted effects suggested several interesting associations, with potential for revealing biological processes relevant to the studied quantitative traits. This work shows that genomic selection can be successfully applied in biomass sorghum breeding programs.

**Electronic supplementary material:**

The online version of this article (10.1007/s11032-018-0802-5) contains supplementary material, which is available to authorized users.

## Introduction

Increasing concerns about environmental issues have aroused global interest in the use of alternative sources for energy production. The use of plant biomass emerges as a viable alternative for the generation of biofuels (Rooney et al. 2007; Morris et al. 2013). Different organic materials have been tested, including high-biomass sorghum (*Sorghum bicolor* L. Moench), which has potential to become an important crop for bioenergy production. This is due to its high biomass content, low water and fertilizer requirements, well-established production systems, tolerance to drought and heat, and high genetic diversity (Murray et al. 2008; Calviño and Messing 2012; Cotton et al. 2013; Brenton et al. 2016). High-biomass sorghum can be used for cellulosic ethanol or bioelectricity production. Cellulosic ethanol, or second generation ethanol, is produced from the hydrolysis of plant biomass into simple sugars, which in turn can be used for ethanol production by fermentation (Sticklen 2008; Zheng et al. 2009; Mandegari et al. 2017). The main focus of bioenergy-targeted breeding programs is to maximize biomass production per land unit, without increasing the energy input, in order to minimize the use of cultivable area. Breeders also aim at modifying cell wall polymers in order to facilitate the subsequent industrial processes. Modifying cell wall composition by, for example, reducing lignin content, may increase the efficiency at which biomass is converted into ethanol (Vermerris et al. 2007; Edmé et al. 2017).

Sorghum is a diploid species (2n = 20) with a relatively small genome, around 700 Mbp (Paterson et al. 2009), for which a reference sequence is already available. This resource facilitates breeding efforts involving molecular markers, because it is possible to confirm previous results and use genome annotations to make inferences concerning potential candidate genes, for example. Sorghum biomass breeding programs can take advantage of methods that reduce the time required to complete a breeding cycle and enable early and efficient selection of superior genotypes. Genomic selection has great potential to attain these goals. This strategy was first proposed by Meuwissen et al. (2001) to increase the efficiency of marker-assisted selection and accelerate the breeding cycle. The method uses markers distributed across the genome to predict the breeding value of individuals. Genomic selection was first applied in an animal breeding context, due to high cost of phenotyping and the impossibility of using replicates (Piyasatian et al. 2006; Solberg et al. 2006; Schaeffer 2006; Dekkers 2007; Long et al. 2007; Lee et al. 2008; Legarra et al. 2008; VanRaden et al. 2009). Later, it also attracted the attention of plant breeders (Bernardo and Yu 2007; Heffner et al. 2009; Jannink et al. 2010). Simulation and empirical studies performed in various crops showed the superiority in terms of accuracy of genomic selection over traditional marker-assisted selection and selection based only on pedigree information (Bernardo and Yu 2007; Bernardo 2009, 2014a; Zhong et al. 2009; Lorenzana and Bernardo 2009; Mayor and Bernardo 2009; Crossa et al. 2010b, 2013, 2014; Grattapaglia and Resende 2010; Zhao et al. 2013).

In recent years, the development of next generation sequencing allowed genome-wide genotyping at lower costs. The genotyping-by-sequencing (GBS) technique is a multiplex system that allows the simultaneous identification of thousands of single-nucleotide polymorphisms (SNPs) and genotyping of the entire population of interest. This strategy has been used for a variety of species, such as barley, wheat, maize, rice, grapes, cocoa, sorghum, bean, soybean, cassava, cranberry, grass, sunflower, and oil palm (Elshire et al. 2011; Hansey et al. 2012; Poland et al. 2012; Sonah et al. 2013; Lu et al. 2013; Morris et al. 2013; Pootakham et al. 2015; Bredeson et al. 2016; Celik et al. 2016; Covarrubias-Pazaran et al. 2016; McAllister and Miller 2016). Due to the large number of markers, this technique is suitable for genomic selection (Poland and Rife 2012).

Various statistical models have been proposed for use in genomic selection (Meuwissen et al. 2001; Gianola et al. 2003; Park and Casella 2008; Habier et al. 2011). The main distinction between these models is the assumption about the underlying distribution of quantitative trait loci (QTL) effects. Due to particularities of the genetic architecture of different quantitative traits (Jiang and Zeng 1995; Zeng et al. 1999; Zeng 2001), distinct genomic selection models may be suitable for different phenotypic traits. Genomic selection models estimate the effects of individual markers and use information simultaneously from all markers available to estimate breeding values, without testing for individual marker effects; the aim is the selection directly applied to plant breeding (Bernardo and Yu 2007; Dekkers 2007; Goddard and Hayes 2007; Habier et al. 2007). Nevertheless, it is expected that markers within genes and/or with particular functional roles present effects of higher magnitude. Recently, novel models that exploit prior biological information in the analysis were proposed (Edwards et al. 2016; MacLeod et al. 2016). The predicted effects of markers in genomic selection studies can thus be used for functional enrichment analysis.

This work aimed to predict genomic breeding values of a high-biomass sorghum panel for bioenergy production. In addition, we investigated the potential use of functional enrichment analysis on marker-predicted effects for revealing important biological pathways involved in controlling quantitative traits related to biomass production and quality.

## Materials and methods

### Plant material

In this study, we analyzed a panel of 200 sorghum genotypes from Embrapa Maize and Sorghum. The panel is divided into two equally sized sub-panels. The 100 lines of sub-panel I are composed of materials from Embrapa germplasm bank and core collections from CIRAD and ICRISAT, consisting of 50 lines of high-biomass sorghum and 50 lines of saccharine sorghum. The remaining 100 lines of sub-panel II consist of high-biomass sorghum derived from Embrapa germplasm bank, originated mostly from accessions from the USA and some from Africa. These were added to Embrapa’s breeding program and later to the panel and were characterized by lower genetic variability, higher sensitivity to photoperiod, and high levels of cellulose when compared to genotypes of sub-panel I. Each sorghum line in the panel is identified in Supplementary Table 1.

### Molecular data

The 200 lines were genotyped using the GBS technology. We applied the standard GBS protocol (Elshire et al. 2011) with restriction enzyme ApeKI and 96-plex approach. We then used the BWA (Li and Durbin 2009) to align tags to the reference genome of *Sorghum bicolor* (v2.1) and TASSEL-GBS (Glaubitz et al. 2014) pipeline for SNP calling.

We initially assessed the quality of these genotypic data with the following statistics: frequency of heterozygous genotypes for each SNP, minor allele frequency (MAF), and frequency of missing data for each genotype and each SNP. Missing marker values were then imputed using the NPUTE software (Roberts et al. 2007). After the imputation, we discarded markers with MAF lower than 5% and recoded the genotypes, assigning a value of −1 or 1 to the two possible homozygote genotypes.

### Population structure

We performed principal component analysis (PCA) to evaluate population structuring in the panel, using the R package pcaMethods (Stacklies et al. 2007). We also inferred population structure with STRUCTURE 2.3.4 (Pritchard et al. 2000). To warrant the use of unlinked markers, the genetic data were pruned to remove SNPs with an *r*^2^ value higher than 0.20, in sliding windows of 2 Mbp, using the SPNRelate R package (Zheng et al. 2012). Using a threshold value of 0.2 eliminates a large degree of redundancy in the data and reduces the influence of chromosomal artifacts (Laurie et al. 2010). The linkage disequilibrium pruning step left 23,117 SNPs, which were used to perform the structure analysis. We tested number of populations (*K*) varying from one to ten, running a series of ten independent runs for each value of K. Each run consisted of a burn-in period of 100,000 and 200,000 MCMC iterations. For the choice of the most likely value of *K*, we used the *∆K* method, described by Evanno et al. (2005) and implemented in the Structure harvester software (Dent and Bridgett 2012). The most likely value of *K* was used to obtain conditional population membership coefficients of each individual.

### Phenotypic data

The 100 lines of sub-panel I were evaluated phenotypically for two years (2011 and 2012) and the 100 lines of sub-panel II for one year (2011), in Sete Lagoas, Minas Gerais State, Brazil. Summary statistics of these data are presented in supplementary material (Supplementary Tables 2 and 3; Supplementary Figs. 1 and 2). The experimental design consisted of a lattice (10 × 10) with three replicates. Plots were composed by 5 m lines spaced at 0.7 m, and showing nine plants per linear meter density. We evaluated the following phenotypic traits: days to flowering, number of days from seeding to the beginning of the pollen release in 50% of the plants in each plot; plant height, average height in meters of all plants in each plot, measured from the soil surface to the apex of the panicle; fresh matter yield (FMY) and dry matter yield (DMY), given in kg/plot by weighing all plants (whole) of each plot, harvested at grain physiological maturity, and then converted to t ha^−1^. To determine the dry matter, we took samples of fresh matter from the plot, which were incubated in an oven at 65 °C for 72 h or until sample weight was constant. Subsequently, by the difference between the dry and fresh weights, we obtained the percentage of dry matter for each plot. Additionally, biomass samples were characterized by determining acid detergent fiber (ADF), neutral detergent fiber (NDF), cellulose, hemicellulose and lignin (acid detergent lignin), according to Van Soest et al. (1991), and their values are presented as a percentage of dry matter weight.

### Phenotypic analyses

We initially fitted appropriate mixed models for the phenotypic data of sub-panel I and sub-panel II. The statistical model for each trait of sub-panel I was as follows:

1 $$ {y}_{\mathrm{i} jkm}=\mu +\beta {s}_{ikm}+\gamma {d}_{ikm}+{a}_m+{r}_{km}+{b}_{jkm}+{g}_{im}+{\varepsilon}_{ijkm} $$

where *y*_*ijkm*_ is the phenotype of the *i*th genotype of block *j*, in replicate *k* and year *m*; *μ* is the intercept; *s*_*ikm*_ is the crop stand of the *i*th genotype, in replicate *k*, year *m*, corresponding to the count of plants that were effectively evaluated during the harvest period, and *β* is the corresponding fixed effect; *d*_*ikm*_ is a covariate representing the number of days to flowering for the *i*th genotype, in replicate *k*, year *m*, and *γ* is the corresponding fixed effect; *a*_*m*_ is the fixed effect of year *m*; *r*_*km*_ is the random effect of replicate *k* in year *m*; *b*_*jkm*_ is the random effect of block *j*, in replicate *k*, in year *m*; *g*_*im*_ is the random effect of the *i*th genotype in year *m*; and *ε*_*ijkm*_ is a random non-genetic effect. The correction for the effect of days to flowering aimed to eliminate the influence of early or late flowering on other traits. Particularly for biomass production, this allows selecting genotypes that contribute with high biomass alleles independently of their flowering behavior. This correction was included when fitting the model for all traits except days to flowering.

The statistical model for each trait of sub-panel II can be indicated by the following:

2 $$ {y}_{ijk}=\mu +\beta {s}_{ik}+\gamma {d}_{ik}+{r}_k+{b}_{jk}+{g}_i+{\varepsilon}_{ijk} $$

where *y*_*ijk*_ is the random phenotypic effect of the *i*th genotype of block *j*, in replicate *k*; *s*_*ik*_ is the crop stand of the *i*th genotype in replicate *k*, and *β* is the corresponding fixed effect *d*_*ik*_ is a covariate representing the number of days to flowering for the *i*th genotype, in replicate *k*, and *γ* is the corresponding fixed effect; *r*_*k*_ is the random effect of replicate *k*; *b*_*jk*_ is the random effect of block *j*, in replicate *k*; *g*_*i*_ is the random effect of the *i*th genotype and *ε*_*ijk*_ is a random non-genetic effect.

The model assumes that the random effect of genotype *g*_*i*_ follows a normal distribution with zero mean and variance ${\sigma}_g^2$. For the effects of replicate *r*_*km*_ or *r*_*k*_, block *b*_*jkm*_ or *b*_*jk*_ and non-genetic effects *ε*_*ijkm*_ or *ε*_*ijk*_, we fit different (co)variance structures, including the identity, diagonal, compound symmetry and unstructured models (Smith et al. 2001). The variance-covariance (VCOV) matrices for these purposes have been structured for convenient grouping factors: replicates were used as a grouping factor for the block effect, and blocks within replicates were used as a grouping factor for the residual effects. Year was also used as a grouping factor for all these effects in sub-panel I, including genotype, which allows us to model both the main genotype effect and the genotype by year interaction. We initially compared different models for the VCOV structure of replicate, block and genetic effects, using the Bayesian Information Criterion (BIC; Schwarz 1978). We then evaluated similar structures for the non-genetic effects. Finally, we used the best fitting VCOV structure model to obtain the best linear unbiased predictors (BLUPs) of the genotypes for each analyzed trait (Supplementary Tables 4 and 5).

Fitting model (1) with a compound symmetry structure for the genotype by year interaction allowed the trait heritability to be estimated for sub-panel I, with the following equation:

$$ {h}^2=\frac{\sigma_{\mathrm{g}}^2}{\left({\sigma}_{\mathrm{g}}^2+\frac{\sigma_{\mathrm{g}\mathrm{a}}^2}{m}+\frac{\sigma_{\mathrm{e}}^2}{n_{\mathrm{r}}m}\right)} $$

where *n*_r_ is the number of replicates, *m* is the number of years, ${\sigma}_{\mathrm{g}}^2$ is the genetic variance component, ${\sigma}_{\mathrm{ga}}^2$ is the genotype by year interaction variance component, and ${\sigma}_{\mathrm{e}}^2$ is the residual variance component. Similarly, for sub-panel II, we estimated heritability based on model (2) with the following equation:

$$ {h}^2=\frac{\sigma_{\mathrm{g}}^2}{\left({\sigma}_{\mathrm{g}}^2+\frac{\sigma_{\mathrm{e}}^2}{n_{\mathrm{r}}}\right)} $$

where terms are as previously defined. All the analyses were performed using the software GenStat, version 16 (Payne et al. 2013).

### Fitting of genomic selection models

We used the R packages BGLR (Pérez and de Los Campos 2014) to fit the Bayesian models Bayes A, Bayes B, Bayes C*π*, Bayes Lasso and Bayes RR, and rrBLUP (Endelman 2011) to fit the random regression best linear unbiased predictor (RRBLUP) model. We used 40,000 iterations for Bayesian models, with 20,000 discarded as burn-in, and assumed default parameters for prior models. Our analyses used a cross-validation procedure to evaluate the ability of a model to predict breeding values. To this end, the 200 genotypes were divided into ten mutually exclusive groups, each containing 20 genotypes. For each cross-validation set, we began by fitting the genomic selection models on a training set of 180 genotypes, to estimate marker effects based on genotypic and phenotypic information. These marker effects then provided estimates of the breeding values of the remaining 20 individuals, based only on genotypic information—genomic estimated breeding value (GEBV). Finally, the correlation between the GEBVs and the estimated breeding values, i.e., the BLUPs obtained in the phenotypic analyses, provided estimates of the predictive abilities of the genomic selection models.

We also wanted to investigate the application of genomic selection models, trained on sets of selected individuals and years, to predict the behavior of genotypes across populations and/or years. The models were thus used for prediction across sub-panels, that is, the 100 genotypes of sub-panel I were used as training set and the 100 genotypes of sub-panel II as the test set. First, we used estimated breeding values for sub-panel I based on the combined data from two years. We also investigated the use of data for each year, separately, to assess the prediction across sub-panels and across sub-panels and years, respectively. Finally, we investigated the performance of genomic selection across years, training the models for sub-panel I in the first year to predict the behavior of the same genotypes in the second year.

### Effect of marker density

We evaluated the effect of marker density on the efficiency of genomic selection. Multiple scenarios with reduced numbers of markers were delineated and the RRBLUP model was fitted to each of them, providing estimates of predictive abilities. Starting from the complete set of 258,220 markers, we randomly removed half of the markers at each step, down to a minimum of 16. From the complete set of markers, ten random subsets were obtained for each tested marker density. We only used the RRBLUP model due to its lower computational requirements compared to the other models.

We also evaluated the consequences of removing markers of small effects. For this purpose, we initially fitted the RRBLUP model to all markers. Then, half of the markers with effects of greater magnitude was selected and used again to fit the RRBLUP model. We did this successively until a minimum of 16 markers, always choosing the markers of higher (absolute) effects (Supplementary Fig. 3). We did this selection of markers of larger effects using a ten-fold cross validation strategy as previously described, to avoid bias in the selection of markers.

### Functional enrichment

Given a set of predicted marker effects, we wanted to test the hypothesis that some SNPs with particular biological functions had higher effects on the estimation of breeding values for each trait. To that end, we performed functional enrichment analysis of these marker effects. The sorghum genome obtained from the Phytozome platform (Paterson et al. 2009; Goodstein et al. 2012) includes predicted gene models and annotation of gene ontology (GO) terms for each predicted gene product (Paterson et al. 2009). This functional classification provides an ontology of terms representing the biological properties of a gene product (Ashburner et al. 2000). We assigned each SNP located inside a gene product all GO terms associated with that gene. All markers matching a given GO term found in this data set formed a distinct GO cluster. We then applied the Kolmogorov–Smirnov test (Frank and Massey 1951) to compare the distribution of the effects of all markers in a GO cluster with the distribution of the effects of all the remaining markers, individually for each trait. Our aim was to detect GO terms in which the marker effects were on average higher than the effects of the set of all markers. For this reason we chose a unilateral significance test to compare the absolute values of marker effects. Type I error control for multiple tests was done with the false discovery rate (FDR) correction (Benjamini and Hochberg 1995). GO terms with adjusted *p* value < 0.01 were deemed significant. We conducted the functional enrichment analysis separately for markers effects predicted with all genomic selection models.

## Results

### Genotypic data

Genotyping of the 200 high-biomass sorghum genotypes generated a total of 1,024,892 SNPs, distributed along the ten chromosomes (Supplementary Fig. 4). The proportion of heterozygous genotypes per SNP ranged from 0 to 95%, with mean of 4.2% and median of 2.2% (Supplementary Fig. 5). These loci with high heterozygosity likely corresponded to spurious polymorphisms from duplicated genomic regions (Glaubitz et al. 2014), artifacts which were removed during marker imputation.

An evaluation of the proportion of missing data showed that SNPs had between 0 and 99.5% of missing genotype calls, with mean of 34.6% and median of 28.5% (Supplementary Fig. 6). Because sorghum is a diploid, self-pollinating species with an available reference genome, imputation of missing data is greatly facilitated. After imputation was performed, the distribution of minor allele frequencies showed that most SNPs had MAF lower than 5% (Supplementary Fig. 7). In general, low MAF rates may represent sequencing errors, rare alleles, and low coverage. For this reason, we chose to filter SNPs with a minimum MAF value of 5%, which retained 258,220 SNPs, which were used for the fitting of genomic selection models.

### Population structure

**Fig. 1:**
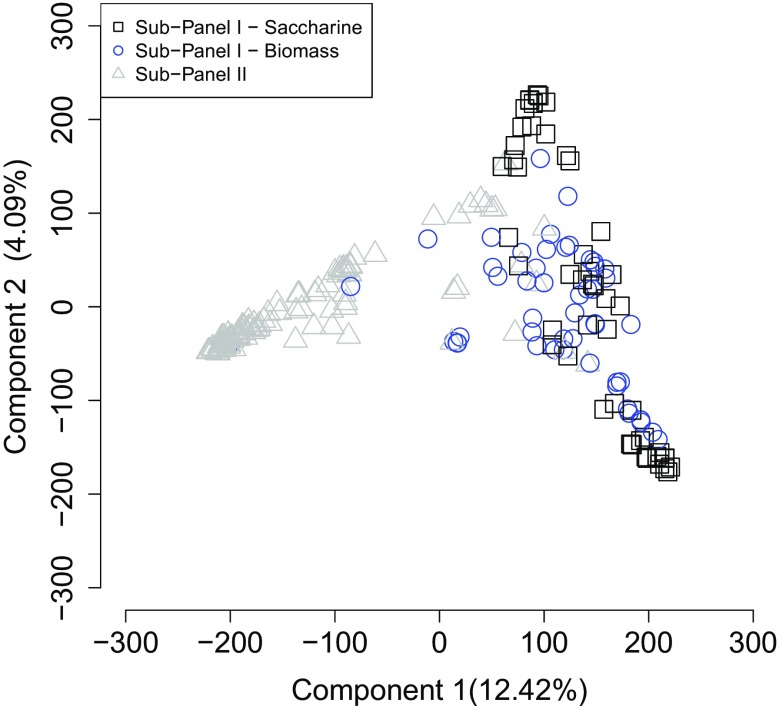
Scatter plot of the two first principal component scores of 200 high-biomass sorghum genotypes. Sorghum genotypes belong to the Embrapa Maize and Sorghum germplasm bank and breeding program. Component scores were obtained from a Principal Component Analysis based on 258,220 SNP markers. Each solid circle represents a genotype, and the colors indicate the sub-panel it belongs

Principal component analysis revealed structuring of genotypes between the two sub-panels. Differences in the first principal component reflected the separation of individuals from sub-panels I and II (Fig. 1). Interestingly, sub-panel I did not show distinctive structuring between saccharine and biomass genotypes, with the former being separated into two main groups, but with substantial overlapping of genotypes. These results are in agreement with those obtained by STRUCTURE, which indicated the best fit was for a value of *K* of two (Supplementary Fig. 8), with membership coefficients of individuals coinciding with the first PCA component (Supplementary Fig. 9).

### Predictive ability

We observed through the estimated correlation between the breeding values predicted by genomic selection and the estimated breeding values that the highest predictive abilities were achieved for traits ADF, NDF, cellulose, and lignin, while traits hemicellulose and days to flowering had the lowest values (Table 1). In general, all predictive abilities obtained were high, ranging from 0.85 for NDF to 0.66 for days to flowering.

**Table 1:** Predictive abilities obtained from six genomic selection models applied to nine traits of the high-biomass sorghum panel of Embrapa Maize and Sorghum in the joint analysis. Values indicate the correlation coefficient between the breeding values predicted by genomic selection models and the phenotypic breeding values

Trait	Heritability		Genomic selection model					
	Sub-panel I	Sub-panel II	BayesB	BayesA	BayesRR	BayesC	BayesLasso	RRBLUP
Plant height	0.96	0.83	0.77	0.77	0.77	0.77	0.76	0.78
Cellulose	0.78	0.89	0.83	0.83	0.83	0.83	0.82	0.83
ADF	0.83	0.86	0.83	0.83	0.83	0.83	0.82	0.84
NDF	0.76	0.88	0.84	0.85	0.84	0.84	0.84	0.85
Days to flowering	0.81	0.87	0.64	0.64	0.64	0.63	0.61	0.66
Hemicellulose	0.39	0.51	0.68	0.68	0.68	0.68	0.67	0.68
Lignin	0.82	0.61	0.82	0.82	0.82	0.82	0.82	0.82
DMY	0.70	0.67	0.73	0.73	0.73	0.73	0.72	0.74
FMY	0.80	0.85	0.77	0.77	0.77	0.77	0.76	0.77

*ADF*, fiber proportions in acid detergent; *NDF*, fiber proportions in neutral detergent; *DMY*, dry matter yield; *FMY*, fresh matter yield

All tested genomic selection models yielded similar predictive abilities for each of the nine traits. Even though differences between models were modest, the RRBLUP model showed the best predictions overall, while the Bayes Lasso model showed the lowest predictive abilities. For example, for the trait plant height, the best and worst models provided values of 0.78 and 0.76, respectively.

We note that the trait days to flowering yielded the lowest predictive abilities, despite having heritabilities of 0.81 and 0.87, for sub-panel I and II, respectively. In contrast, hemicellulose showed lower heritabilities, 0.39 (sub-panel I) and 0.51 (sub-panel II), but yielded a predictive ability of 0.68.

To assess whether these accuracies were influenced by the population structure present between sub-panels, we also performed a validation of predictive accuracy within each sub-panel. The predictive accuracy was high for all traits in both sub-panels, except for the trait hemicellulose in sub-panel II (Supplementary Table 6).

**Fig. 2:**
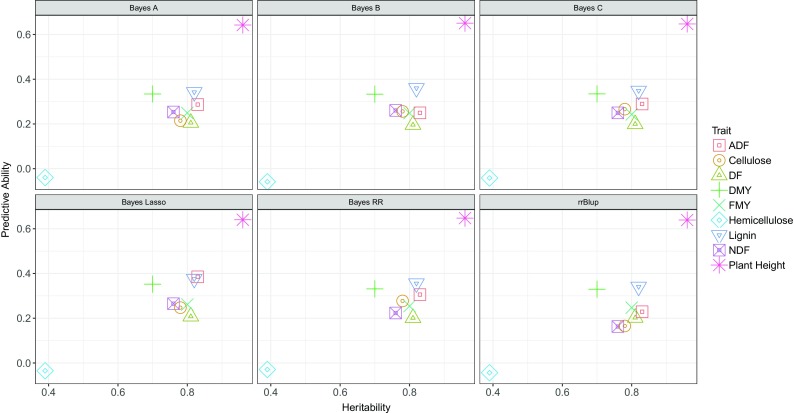
Relationship between trait heritability and predictive ability for different genomic selection models. Models Bayes A, Bayes B, Bayes Cπ, Bayes Lasso, Bayes RR, and RRBLUP were applied to nine traits of the high biomass sorghum panel of Embrapa Maize and Sorghum, for the prediction across sub-panels

When we performed the prediction across sub-panels, using data from both years for sub-panel I, the predictive abilities achieved were considerably lower compared to those obtained in the analysis of the complete panel (Supplementary Table 7). For example, for the trait hemicellulose the predictive ability ranged from 0.68 in the analysis of the complete panel to − 0.04 in the analysis across sub-panels. In this situation we observed a stronger correlation between heritability and predictive abilities (Fig. 2). Interestingly, when we evaluated prediction across sub-panels (Supplementary Table 8), training the models only with data from the first year, we obtained predictive abilities higher in most traits, compared to those obtained in the analysis with both years. Indeed, the use of genomic selection models across sub-panels and years (Supplementary Table 9) resulted in the lowest predictive abilities. Finally, when analyzing the use of models across years for sub-panel I (Supplementary Table 10), observed predictive abilities were high indicating a better performance of genomic selection when the training and test populations were more closely related.

### Marker density

**Fig. 3:**
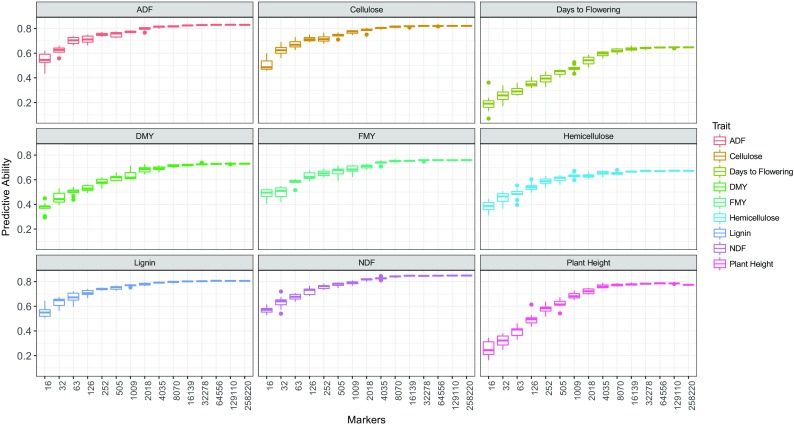
Predictive abilities of the model RRBLUP as a function of marker density for nine traits of the high-biomass sorghum panel

Effect of marker density on the predictive abilities of the model RRBLUP for each of the nine traits is shown in Fig. 3. As the marker density increased, the predictive abilities also increased until reaching a plateau starting with roughly 2018 markers. The predictive abilities were maximum and the variance minimum when using the complete set of markers, for all traits. Conversely, the lowest marker density resulted in the minimum predictive abilities and maximum variance. For example, in the trait plant height, the predictive ability varied from 0.77 with 258,220 markers to 0.25 with 16 markers.

When we fitted the model using the markers with larger effects, we observed little influence on the predictive abilities compared with those obtained with the complete set of markers (Supplementary Fig. 3). Similarly to our observation based on the random selection of markers, predictive accuracies remained high even using one to eight thousand markers. For example, for the trait plant height the correlation varied from 0.80 to 0.78, with 258,220 and 505 markers, respectively. However, for reduced numbers of markers (i.e., less than 1009), the predictive abilities achieved were considerably lower. With the minimum of 16 markers the predictive abilities ranged from 0.53 for the trait hemicellulose to 0.76 for the trait NDF.

### Functional enrichment

Analysis of enrichment of marker effects predicted with different genomic selection models yielded different numbers of enriched GO terms (results not shown). Bayesian models resulted in small numbers of terms, which were also largely identified with the RRBLUP model. For this reason, we choose to only present the results from RRBLUP. We found a total of 1119 GO terms related to the SNPs present inside the predicted gene models. The number of SNPs associated with a single GO term ranged from 1 to 14,625, with mean 214.9 and median 26. The number of GO terms associated with a single SNP ranged from 1 to 40, with mean 3.27 and median 3. The results of functional enrichment analyses based on the Kolmogorov-Smirnov test showed between 58 and 116 significant terms for the traits analyzed (Supplementary Tables 11 to 19). Hemicellulose was associated with the largest number of GO enriched terms, 116. On the other hand, days to flowering showed the smallest number of significant GO terms, 58.

**Fig. 4:**
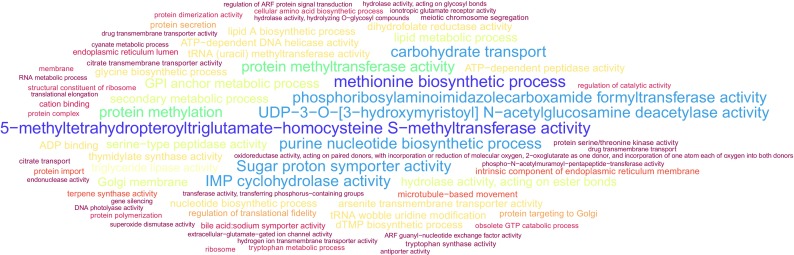
Functional enrichment of Gene Ontology biological process terms of the marker predicted effects for the trait cellulose. The size of the text depends on the *p* value

For most traits, the enriched GO terms were related to the synthesis and metabolism of biomolecules, such as amino acids, fatty acids, nucleotides, proteins, and carbohydrates. We also found terms regarding the secondary metabolism, autophagy, catabolic processes of macromolecules of the cell wall, and cell division (Supplementary Tables 11 to 19). For the trait plant height, we found GO terms related to autophagy and small GTPase-mediated signal transduction. Yield related traits, FMY and DMY, showed enrichment for GO terms related to carbohydrate transport, sugar proton symporter activity and Golgi membrane. For the trait days to flowering we found terms related to protein modification, activation and deactivation of enzymes, and autophagy. Fiber composition traits, ADF and NDF, exhibited terms related to carbohydrates and cellular export. GO terms related to carbohydrates, especially transport, protein anchoring to the plasma membrane and GPI anchor metabolic process were detected among those related to hemicellulose content. Enriched terms for lignin content include those related to exocytosis, anchor and biosynthesis of macromolecules, particularly the aromatic amino acid family biosynthetic process. Finally, Fig. 4 shows that the GO terms highlighted for cellulose content were associated with carbohydrate transport, GPI anchor metabolic process, movement of microtubules, and enzymatic activity.

## Discussion

The emergence of next-generation sequencing technologies enabled the use of genome-wide markers at relatively reduced costs for many species. Among the various genotyping techniques available, the GBS system provides a quick and lower cost tool (Poland and Rife 2012). The large amount of missing data is one of the known disadvantages of this technique (Davey et al. 2011; Beissinger et al. 2013). However, because we used homozygote lines and sorghum is a self-pollinating diploid species with an available reference genome, genotype imputation allowed the use of GBS without losses in predictive ability (Habier et al. 2009; Weigel et al. 2010; Dassonneville et al. 2011; Mulder et al. 2012). Even after filtering out SNPs with MAF lower than 5%, it was possible to obtain dense genome coverage. The 258,220 SNPs correspond to approximately one SNP every 2.8 Kbp.

It is interesting that several genomic selection models are adjusted and compared, such that selection can be made based on the best model for each trait. However, limitations of computational resources and time can prohibit such comparison and require the application of a simpler model. Although each compared genomic selection model has different assumptions about the underlying distribution of the QTL effects, the differences between their predictive abilities were very small (Table 1). In a study with maize, Bernardo and Yu (2007) reported that Bayesian models exhibited little or even no advantage when compared to the RRBLUP model. Due to its lower computational complexity, the ordinary application of RRBLUP in breeding programs can be considered a viable alternative (Hofheinz and Frisch 2014). However, it can still be relevant to compare different models, because successive application of genomic selection through breeding cycles may affect the outcome of models with different assumptions (Habier et al. 2007).

Phenotyping is very important in genomic selection studies, since it impacts all steps of the process, from the prediction of markers effects to the selection of superior genotypes. Thus, the precision with which phenotypic measurements are taken for each trait influences the predictive abilities of the models (Heslot et al. 2015). Days to flowering is an extremely important trait for breeding programs of high-biomass sorghum. Indeed, the genetic characterization of a regulatory system responsible for controlling the photoperiod and flowering time in sorghum enabled the development of high-biomass hybrids. These hybrids are highly sensitive to photoperiod and with late flowering, which exhibit prolonged vegetative growth and high biomass accumulation (Rooney and Aydin 1999; Rooney et al. 2007; Murphy et al. 2011). For this reason, we included a covariate to adjust for differences in days to flowering between genotypes.

The size and composition of the training population are important factors that can be manipulated by breeders (Habier et al. 2010; Rincent et al. 2012). The combination of training datasets maximizes the use of phenotypic and genotypic information available, generating large training populations and increasing the predictive ability (De Roos et al. 2009; Hayes et al. 2009; Asoro et al. 2011; Lorenz et al. 2012; Technow et al. 2013). In our study, we conducted joint analysis of the two sub-panels to ensure that a larger training population was used. Hence, the fact that the predictive abilities found (Table 1) were higher than those obtained in the cross analysis between sub-panels (Supplementary Table 6) can be partly explained by the size of the training population in each scenario. It is also expected that genetic predictions are more accurate for traits with high heritability (Combs and Bernardo 2013; Lorenz 2013). We observed such a trend in the analysis between sub-panels (Fig. 2). However, in the joint analysis we did not observe a strong trend between heritability and prediction accuracy (Table 1). Several authors showed that predictive abilities are also affected by effective population size, training population size, linkage disequilibrium, trait architecture, marker density, choice of predictive model and the relationship between training and breeding population (Daetwyler et al. 2008; Grattapaglia and Resende 2010; Asoro et al. 2011; Nakaya and Isobe 2012; de los Campos et al. 2013). In this way, the fact that the prediction ability for plant height was not as high as its heritability could suggest that the predictive ability of this trait was affected by other factors. Similarly, the days to flowering trait had one of the lowest predictive abilities, despite showing high heritability. This lack of correlation between predictive ability and heritability is in agreement with other results in the literature (Grattapaglia and Resende 2010; Heffner et al. 2011).

One of the questions that arise in genomic selection studies is that the training and testing populations must be highly related to ensure an effective selection. The interaction of trait architecture and population structure plays an important role in creating a training population. Our principal component analysis (Fig. 1) showed that the structure of the 200 genotypes of the panel reflect their subdivision in two sub-panels. Sub-panel I has mostly saccharine and biomass genotypes from CIRAD and ICRISAT, while sub-panel II is mainly composed of genotypes from the Embrapa sorghum germplasm bank and breeding program. In the joint analysis, the similarity between genotypes in training and test populations was large. This can partially explain the fact that the predictive abilities found in the joint analysis (Table 1) were higher than those obtained in the cross analysis between sub-panels (Supplementary Table 7). Nonetheless, it is interesting to note that predictive abilities were still moderately high for plant height. In addition to the influence of population structure on accuracy, we observed a negative impact of genotype by year interaction on the use of genomic selection between sub-panels. Indeed, the predictive ability between sub-panels was higher when we only used data from the first year for sub-panel I, and inclusion of data from the second year reduced accuracy (Supplementary Tables 7 and 8).

The effects of marker density on the predictive abilities showed that the best predictions were obtained using the maximum marker density (Fig. 3). The median predictive abilities remained relatively constant with reduced numbers of markers, down to roughly four thousand markers. From a practical standpoint, this indicates that a reduced number of markers explained most of the genetic variation, opening new perspectives for the use of a relatively small subset of SNPs in sorghum, as is done for other plant and animal species, by constructing genotyping chips (Matukumalli et al. 2009; Yu et al. 2013; Wang et al. 2014). In any case, this may not be true for other breeding scenarios, and using the complete set of markers resulted in the highest accuracies (Meuwissen and Goddard 2010).

Genomic selection has revolutionized the use of marker-assisted selection in plant breeding, mainly due to its distinct approach when compared to QTL mapping and genome-wide association studies (GWAS). Standard QTL mapping aims to map chromosomal regions affecting phenotypic traits of interest, thus enabling the use of markers linked to these regions (Bernardo 2008; Lorenz et al. 2011). However, the use of QTL mapping in breeding programs is limited by the fact that the commonly used bi-parental populations have applications that may be conditioned to the specific population under study. Besides that, the statistical models used are unsuitable for breeding of polygenic traits, which are controlled by many loci of small effect (Meuwissen et al. 2001; Goddard and Hayes 2007; Heffner et al. 2009). The main objective of GWAS is also the identification of chromosomal regions associated with a particular trait, using a diversity panel instead of a breeding population (Ingvarsson and Street 2011; Huang and Han 2014). In contrast, genomic selection does not intend to test for the significance of genes and/or individual markers. It instead leverages information from all available genome-wide markers. This makes this methodology directly applicable to plant breeding (Bernardo and Yu 2007; Dekkers 2007; Goddard and Hayes 2007; Habier et al. 2007).

Nevertheless, we expect that markers located near genes responsible for certain biological functions present effects of greater magnitude. Thus, the predicted effects of markers in genomic selection studies can be used for functional enrichment analysis to identify particularly important functional groups. We stress that this strategy only considered markers located within genes, but our results indicate that 28.4% of the SNPs were located in functionally annotated genes. Our data revealed several interesting associations for the several traits evaluated, which should be further investigated. As an example, the detection of GO terms related to microtubule-based movement for the trait cellulose (Fig. 4) is possibly connected with the fact that the deposition of cellulose is guided by microtubules that are adjacent or directly connected to a synthesis complex (Delmer and Amor 1995). Besides, in agreement with published studies (Gillmor et al. 2005; Ben-Tov et al. 2015), GO terms related to GPI anchor metabolic process are expected to be associated with cellulose deposition. We also observed several carbohydrate-related GO terms for hemicellulose, such as carbohydrate transport, Golgi membrane, GPI-anchor metabolism, and cell wall modification. After hemicellulose is synthesized in the Golgi complex, it is transported to the plasma membrane, so the identification of these GO terms is expected (Pauly et al. 2013). Lignin biosynthesis is initiated in the cytosol with the synthesis of glycosylated monolignols from phenylalanine, an aromatic amino acid. Interestingly, the GO term related to the aromatic amino acid family biosynthetic process is among those enriched for lignin (Boerjan et al. 2003). For the traits days to flowering and plant height, we observed the enriched GO term autophagy. In plant cells, autophagy plays roles in recycling of proteins and metabolites including lipids, and is involved in many physiological processes, such as abiotic and biotic stress response. In addition, autophagy has particular importance on male reproductive development during pollen maturation (Hanamata et al. 2014). Overall, multiple terms related to the biosynthesis of macromolecules were detected for several of the biomass related traits evaluated. This indicates that metabolic processes involving primary metabolites can be important for predicting breeding values. In that case, the selection of genotypes according to their GEBVs may exert stronger selective pressure on these SNPs, because their effects were of greater magnitude. By using this functional enrichment approach, we attempted to couple the application of genomic selection for breeding purposes with the association of particular functional classes of markers with the phenotypic traits. Brenton et al. (2016) performed genome-wide association analysis in a sorghum panel composed of sweet and biomass types and identified potential genes that could lead to bioenergy sorghum improvement. Interestingly, these authors identified a region on chromosome 6 associated with NDF, that had two genes coding for cellulase enzymes, Sobic.006G122200 and Sobic.006G122300. These gene products are responsible for hydrolyzing glycosidic bonds in complex carbohydrates. We also found terms related to the hydrolysis of O-glycosyl compounds (GO:0004553) and carbohydrate metabolic process (GO:0005975) in the enriched gene ontology terms for the trait NDF.

One of the main advantages of genomic selection in breeding programs is the reduced time needed to develop new materials. Genomic selection can reduce the breeding cycle through early prediction of phenotypic performance of a set of genotypes for various traits of interest (Meuwissen et al. 2001; Bernardo and Yu 2007; Bernardo 2008; Crossa et al. 2011). It can also be used to predict phenotypic performance of genotypes for traits of difficult evaluation, such as those related to biomass composition (ADF, NDF, and lignin), which require expensive and laborious phenotypic evaluations. Genomic selection studies in plants have been based on breeding populations, real or simulated (Bernardo and Yu 2007; Crossa et al. 2010a; Zhao et al. 2013). However, in this study we used collections of genotypes with large genetic variability, which constitute diversity panels. Using these panels, we aimed to reduce the time required to select genotypes of biomass sorghum in early stages of Embrapa breeding program, through prediction based on genotypes already selected in other breeding programs (Supplementary Table 6). We achieved predictive abilities in the order of 0.39, 0.38, 0.35, 0.28, 0.26, 0.26, and 0.21 for the traits ADF, lignin, DMY, cellulose, NDF, FMY, and days to flowering, respectively, while for plant height we observed a predictive ability of 0.65. However, for the trait hemicellulose, the predictive abilities found were low and negative. This might be due to the compositional analysis method used to measure hemicellulose content, which is faster and less costly than chromatography-based methods, but also less accurate.

Currently, several studies that apply genomic selection in plant species have been developed (Bernardo and Yu 2007; Bernardo 2009, 2014b; Heffner et al. 2009; Mayor and Bernardo 2009; Jannink et al. 2010 ; Grattapaglia and Resende 2010; Poland et al. 2012; Zhao et al. 2013; Crossa et al. 2013, 2014; Zhang et al. 2014). Using biomass sorghum, Yu et al. (2016), showed the potential use of genomic selection to improve the process of germplasm evaluation in global gene banks. This innovative way to apply this strategy could facilitate downstream breeding and genetic research. With a different approach, our work shows that genomic selection can be successfully applied directly in biomass sorghum breeding programs, which have the potential to help sorghum become an important bioenergy feedstock in Brazil. Yu et al. (2016) applied genomic selection to study eight biomass related traits, including dry biomass yield and plant height. Using a cross-validation scheme they found predictive abilities that ranged from 0.35 to 0.78. Similarly, in our work we found high predictive abilities for the several traits evaluated, contributing to an early and efficient selection of the best genotypes. The models of genomic selection used herein yielded satisfactory results, which are directly applicable to breeding and potentially able to reduce the time required for the launching of new cultivars of biomass sorghum, increasing the potential for this important bioenergy crop. Finally, our functional enrichment analysis attempts to show that, although genomic selection is not primarily focused on identifying and testing markers associated with phenotypes, its results can help in understanding the biological processes involved in the expression of quantitative traits.

## Electronic supplementary material

ESM 1 (DOCX 18 kb)

ESM 2 (18 kb)

ESM 3 (DOCX 18 kb)

ESM 4 (DOCX 15 kb)

ESM 5 (DOCX 14 kb)

ESM 6 (DOCX 17 kb)

ESM 7 (DOCX 21 kb)

ESM 8 (DOCX 21 kb)

ESM 9 (DOCX 19 kb)

ESM 10 (DOCX 20 kb)

ESM 11 (DOCX 22 kb)

ESM 12 (DOCX 18 kb)

ESM 13 (DOCX 23 kb)

ESM 14 (DOCX 19 kb)

ESM 15 (DOCX 21 kb)

ESM 16 (DOCX 22 kb)

ESM 17 (DOCX 7600 kb)

ESM 18 (DOCX 1283 kb)

ESM 19 (DOCX 226 kb)

ESM 20 (DOCX 23 kb)

ESM 21 (DOCX 23 kb)

ESM 22 (DOCX 23 kb)

ESM 23 (DOCX 21 kb)

ESM 24 (DOCX 67 kb)

ESM 25 (DOCX 327 kb)
