# Supplementary material for: Genomic prediction applied to high-biomass sorghum for bioenergy production
Source: Mol Breed. 2018 Apr 10;38(4):49. doi: 10.1007/s11032-018-0802-5 (PMC5893689; doi:10.1007/s11032-018-0802-5)
Supplement: Supplementary file 1 — (DOCX 18 kb) [file 11032_2018_802_MOESM1_ESM.docx]

**Online Resource 1**

**Article Title:** Genomic prediction applied to high biomass sorghum for bioenergy production

**Journal:** Molecular Breeding

**Authors:** Amanda Avelar de Oliveira; Maria Marta Pastina; Vander Filipe de Souza; Rafael Augusto da Costa Parrella; Roberto Willians Noda; Maria Lúcia Ferreira Simeone; Robert Eugene Schaffert; Jurandir Vieira de Magalhães; Cynthia Maria Borges Damasceno; Gabriel Rodrigues Alves Margarido.

**Name, affiliation, and email of corresponding author:**

Gabriel Rodrigues Alves Margarido

Escola Superior de Agricultura Luiz de Queiroz, USP

Piracicaba, SP 13418-900, Brazil

e-mail: gramarga@usp.br

Cynthia Maria Borges Damasceno

Embrapa Milho e Sorgo

Sete Lagoas, MG 35701-970, Brazil

e-mail: [cynthia.damasceno@embrapa.br](mailto:cynthia.damasceno@embrapa.br)

**Supplementary Table 1** Identifiers of the 200 lines of the high biomass sorghum panel

| **Sub-panel** | **Code^a^** | **Line ID** |
| --- | --- | --- |
| I | IND1P1 | 9929030R |
| I | IND2P1 | 9929034R |
| I | IND3P1 | ATF053B |
| I | IND4P1 | ATF054B |
| I | IND5P1 | ATLAS |
| I | IND6P1 | BR001B |
| I | IND7P1 | BR001B bmr6 |
| I | IND8P1 | BR007B |
| I | IND9P1 | BR007B bmr6 |
| I | IND10P1 | BR008B |
| I | IND11P1 | BR008B bmr6 |
| I | IND22P1 | BR506 |
| I | IND12P1 | BRANDES (BR501) |
| I | IND13P1 | BRAWLEY |
| I | IND14P1 | CMSXS156B |
| I | IND24P1 | CMSXS156B bmr6 |
| I | IND15P1 | CMSXS157B |
| I | IND25P1 | CMSXS157B bmr6 |
| I | IND16P1 | CMSXS205B |
| I | IND17P1 | CMSXS205B bmr6 |
| I | IND23P1 | CMSXS211B |
| I | IND18P1 | CMSXS222B |
| I | IND19P1 | CMSXS225B |
| I | IND20P1 | CMSXS226B |
| I | IND21P1 | CMSXS227B |
| I | IND26P1 | COLLIER |
| I | IND27P1 | DALE (BR504) |
| I | IND28P1 | ELLIS SORGO |
| I | IND29P1 | CMSXS651 (Fartura) |
| I | IND30P1 | GEORGIA BLUE RIBBON |
| I | IND31P1 | HODO |
| I | IND32P1 | HONEY |
| I | IND33P1 | ICEBERG |
| I | IND34P1 | IS11119 |
| I | IND35P1 | IS12531 |
| I | IND36P1 | IS14351 |
| I | IND37P1 | IS14414 |
| I | IND38P1 | IS21622 |
| I | IND39P1 | IS21849 |
| I | IND40P1 | IS22294 |
| I | IND47P1 | IS2263 |
| I | IND48P1 | IS23178 |
| I | IND41P1 | IS23645 |
| I | IND42P1 | IS23666 |
| I | IND43P1 | IS23777 |
| I | IND44P1 | IS29233 |
| I | IND45P1 | IS4821 |
| I | IND46P1 | IS7889 |
| I | IND49P1 | KELLERS CRYSTAL DRIP |
| I | IND50P1 | CMSXS650 (Lavrense) |
| I | IND51P1 | McLean |
| I | IND53P1 | MN 1030 |
| I | IND54P1 | MN 1056 |
| I | IND55P1 | MN 1060 |
| I | IND52P1 | MN 1500 |
| I | IND56P1 | MN 1996 |
| I | IND57P1 | MN 4004 |
| I | IND58P1 | MN 4008 |
| I | IND59P1 | MN 4080 |
| I | IND61P1 | MN 4291 |
| I | IND62P1 | MN 4418 |
| I | IND60P1 | MN 4423 |
| I | IND63P1 | MN 4490 |
| I | IND64P1 | MN 4508 |
| I | IND65P1 | MN 4509 |
| I | IND66P1 | MN 4512 |
| I | IND67P1 | MN 4514 |
| I | IND68P1 | MN 4578 |
| I | IND69P1 | MN 4581 |
| I | IND70P1 | MN 752 |
| I | IND71P1 | MN 960 |
| I | IND72P1 | RAMADA |
| I | IND73P1 | REX |
| I | IND74P1 | RIBBON CANE STRAIGHT NECK |
| I | IND75P1 | RIO (BR500) |
| I | IND76P1 | ROMA (BR502) |
| I | IND77P1 | SACALINE |
| I | IND88P1 | CMSXS652 (Santa Eliza) |
| I | IND78P1 | SART |
| I | IND79P1 | SOURLLES |
| I | IND80P1 | SSM029 |
| I | IND81P1 | SSM1284 |
| I | IND86P1 | SSM215 |
| I | IND82P1 | SSM249 |
| I | IND83P1 | SSM275 |
| I | IND84P1 | SSM379 |
| I | IND85P1 | SSM973 |
| I | IND87P1 | SUGAR DRIP |
| I | IND89P1 | THEIS (BR503) |
| I | IND90P1 | TRACY |
| I | IND91P1 | TX2784R |
| I | IND92P1 | TX2784R bmr6 |
| I | IND93P1 | TX635B |
| I | IND94P1 | TX635B bmr6 |
| I | IND95P1 | TX636B |
| I | IND96P1 | WHITE AFRICAN |
| I | IND97P1 | WILEY |
| I | IND99P1 | MN1357 (WILLIANS MISSISSIPI) |
| I | IND98P1 | Willians (WILLIANS TEXAS) |
| I | IND100P1 | WRAY (BR505) |
| II | IND98P2 | MN 1707 |
| II | IND99P2 | MN 1708 |
| II | IND8P2 | IS 16327 - PU 399469 |
| II | IND100P2 | TAM 428 |
| II | IND27P2 | IS 7367 |
| II | IND2P2 | IS 7373 |
| II | IND13P2 | IS 3836 |
| II | IND14P2 | IS 5420 |
| II | IND16P2 | IS 7204 |
| II | IND17P2 | IS 7235 |
| II | IND19P2 | IS 7263 |
| II | IND21P2 | IS 7312 |
| II | IND22P2 | IS 7332 |
| II | IND41P2 | IS 7439 |
| II | IND44P2 | IS 7465 |
| II | IND61P2 | IS 7625 |
| II | IND83P2 | IS 7818 |
| II | IND32P2 | IS 7383 |
| II | IND33P2 | IS 7399 |
| II | IND34P2 | IS 7411 |
| II | IND35P2 | IS 7413 |
| II | IND36P2 | IS 7418 |
| II | IND37P2 | IS 7419 |
| II | IND38P2 | IS 7426 |
| II | IND43P2 | IS 7456 |
| II | IND45P2 | IS 7469 |
| II | IND49P2 | IS 7548 |
| II | IND50P2 | IS 7554 |
| II | IND51P2 | IS 7556 |
| II | IND52P2 | IS 7566 |
| II | IND53P2 | IS 7586 |
| II | IND54P2 | IS 7590 |
| II | IND55P2 | IS 7592 |
| II | IND65P2 | IS 7651 |
| II | IND66P2 | IS 7659 |
| II | IND67P2 | IS 7669 |
| II | IND80P2 | IS 7791-2 |
| II | IND85P2 | IS 7829 |
| II | IND97P2 | IS 9228 |
| II | IND56P2 | IS 7594 |
| II | IND57P2 | IS 7600 |
| II | IND58P2 | IS 7609 |
| II | IND18P2 | IS 7250 |
| II | IND20P2 | IS 7290 |
| II | IND1P2 | IS 7302 |
| II | IND23P2 | IS 7333 |
| II | IND24P2 | IS 7336 |
| II | IND25P2 | IS 7340 |
| II | IND26P2 | IS 7354 |
| II | IND28P2 | IS 7368 |
| II | IND29P2 | IS 7372 |
| II | IND30P2 | IS 7378 |
| II | IND31P2 | IS 7381 |
| II | IND39P2 | IS 7428 |
| II | IND40P2 | IS 7437 |
| II | IND3P2 | IS 7445 |
| II | IND42P2 | IS 7455 |
| II | IND46P2 | IS 7477 |
| II | IND47P2 | IS 7523 |
| II | IND48P2 | IS 7531 |
| II | IND4P2 | IS 7558 |
| II | IND59P2 | IS 7615 |
| II | IND60P2 | IS 7623 |
| II | IND62P2 | IS 7627 |
| II | IND63P2 | IS 7630 |
| II | IND64P2 | IS 7642 |
| II | IND68P2 | IS 7670 |
| II | IND69P2 | IS 7676 |
| II | IND70P2 | IS 7682 |
| II | IND71P2 | IS 7688 |
| II | IND72P2 | IS 7707 |
| II | IND73P2 | IS 7709 |
| II | IND74P2 | IS 7730 |
| II | IND75P2 | IS 7731 |
| II | IND76P2 | IS 7733 |
| II | IND77P2 | IS 7736 |
| II | IND78P2 | IS 7755 |
| II | IND79P2 | IS 7761 |
| II | IND81P2 | IS 7805 |
| II | IND82P2 | IS 7808 |
| II | IND84P2 | IS 7826 |
| II | IND86P2 | IS 7830 |
| II | IND87P2 | IS 7864 |
| II | IND88P2 | IS 7875 |
| II | IND89P2 | IS 7889 |
| II | IND90P2 | IS 7890 |
| II | IND91P2 | IS 7901 |
| II | IND92P2 | IS 7927 |
| II | IND93P2 | IS 7946 |
| II | IND94P2 | IS 7957 |
| II | IND95P2 | IS 7969 |
| II | IND96P2 | IS 7999 |
| II | IND15P2 | IS 6740 |
| II | IND5P2 | IS 10799 |
| II | IND6P2 | IS 10824 |
| II | IND7P2 | IS 10930 |
| II | IND9P2 | IS 18360 |
| II | IND10P2 | IS 22375 |
| II | IND11P2 | IS 22382 |
| II | IND12P2 | IS 22415 |

^a^Code used in the population structure results plot (Fig. S8)
