# Supplementary material for: Genomic prediction applied to high-biomass sorghum for bioenergy production
Source: Mol Breed. 2018 Apr 10;38(4):49. doi: 10.1007/s11032-018-0802-5 (PMC5893689; doi:10.1007/s11032-018-0802-5)
Supplement: Supplementary file 2 — (18 kb) [file 11032_2018_802_MOESM2_ESM.docx]

**Online Resource 2**

**Article Title:** Genomic prediction applied to high biomass sorghum for bioenergy production

**Journal:** Molecular Breeding

**Authors:** Amanda Avelar de Oliveira; Maria Marta Pastina; Vander Filipe de Souza; Rafael Augusto da Costa Parrella; Roberto Willians Noda; Maria Lúcia Ferreira Simeone; Robert Eugene Schaffert; Jurandir Vieira de Magalhães; Cynthia Maria Borges Damasceno; Gabriel Rodrigues Alves Margarido.

**Name, affiliation, and email of corresponding author:**

Gabriel Rodrigues Alves Margarido

Escola Superior de Agricultura Luiz de Queiroz, USP

Piracicaba, SP 13418-900, Brazil

e-mail: gramarga@usp.br

Cynthia Maria Borges Damasceno

Embrapa Milho e Sorgo

Sete Lagoas, MG 35701-970, Brazil

e-mail: [cynthia.damasceno@embrapa.br](mailto:cynthia.damasceno@embrapa.br)

**Supplementary Table 2** Summary statistics for the nine phenotypic traits evaluated in the high biomass sorghum panel. Results are presented separately by sub-panel. Plant height is presented in meters, fresh matter yield (FMY) and dry matter yield (DMY) in t.ha^-1^, acid detergent fiber (ADF), neutral detergent fiber (NDF), cellulose, hemicellulose and lignin are shown as percentages of dry matter yield

| Sub-Panel | Trait | Minimum | Maximum | Median | Mean |
| --- | --- | --- | --- | --- | --- |
| I | Days to Flowering | 54 | 107 | 75 | 75.72 |
|  | Plant Height | 0.7 | 4.970 | 2.55 | 2.42 |
|  | FMY | 12.33 | 140.82 | 51.51 | 55.07 |
|  | DMY | 2.74 | 52.39 | 13.1 | 14.61 |
|  | ADF | 19.99 | 58.58 | 42.08 | 41.58 |
|  | NDF | 29.88 | 85.43 | 68.14 | 67.14 |
|  | Cellulose | 18.18 | 52.12 | 36.23 | 35.70 |
|  | Hemicellulose | 16.95 | 55.31 | 25.54 | 25.95 |
|  | Lignin | 0.77 | 10.51 | 5.88 | 5.86 |
| II | Days to Flowering | 40 | 100 | 79 | 78.8 |
|  | Plant Height | 1.1 | 4 | 2.5 | 2.46 |
|  | FMY | 6.31 | 57.11 | 18.87 | 20.28 |
|  | DMY | 1.51 | 16.64 | 6.27 | 6.83 |
|  | ADF | 31.85 | 60.04 | 49.2 | 48.75 |
|  | NDF | 53.94 | 86.24 | 76.77 | 76 |
|  | Cellulose | 27.36 | 49.06 | 41.27 | 40.97 |
|  | Hemicellulose | 20.49 | 33.45 | 27.09 | 27.25 |
|  | Lignin | 3.11 | 14.32 | 7.88 | 7.78 |
