# Supplementary material for: Genomic prediction applied to high-biomass sorghum for bioenergy production
Source: Mol Breed. 2018 Apr 10;38(4):49. doi: 10.1007/s11032-018-0802-5 (PMC5893689; doi:10.1007/s11032-018-0802-5)
Supplement: Supplementary file 3 — (DOCX 18 kb) [file 11032_2018_802_MOESM3_ESM.docx]

**Online Resource 3**

**Article Title:** Genomic prediction applied to high biomass sorghum for bioenergy production

**Journal:** Molecular Breeding

**Authors:** Amanda Avelar de Oliveira; Maria Marta Pastina; Vander Filipe de Souza; Rafael Augusto da Costa Parrella; Roberto Willians Noda; Maria Lúcia Ferreira Simeone; Robert Eugene Schaffert; Jurandir Vieira de Magalhães; Cynthia Maria Borges Damasceno; Gabriel Rodrigues Alves Margarido.

**Name, affiliation, and email of corresponding author:**

Gabriel Rodrigues Alves Margarido

Escola Superior de Agricultura Luiz de Queiroz, USP

Piracicaba, SP 13418-900, Brazil

e-mail: gramarga@usp.br

Cynthia Maria Borges Damasceno

Embrapa Milho e Sorgo

Sete Lagoas, MG 35701-970, Brazil

e-mail: [cynthia.damasceno@embrapa.br](mailto:cynthia.damasceno@embrapa.br)

**Supplementary Table 3** Summary statistics for the nine phenotypic traits evaluated in the sub-panel I. Results are presented separately by sorghum type. Plant height is presented in meters, fresh matter yield (FMY) and dry matter yield (DMY) in t.ha^-1^, acid detergent fiber (ADF), neutral detergent fiber (NDF), cellulose, hemicellulose and lignin are shown as percentages of dry matter yield

| Type | Trait | Minimum | Maximum | Median | Mean |
| --- | --- | --- | --- | --- | --- |
| Saccharine | Days to Flowering | 61 | 93 | 75 | 74.69 |
|  | Plant Height | 1.10 | 4.75 | 2.60 | 2.67 |
|  | FMY | 26.76 | 128.19 | 54.35 | 58.36 |
|  | DMY | 5.09 | 50.66 | 13.63 | 15.05 |
|  | ADF | 19.99 | 58.28 | 39.68 | 39.66 |
|  | NDF | 29.88 | 82.80 | 64.17 | 64.33 |
|  | Cellulose | 18.18 | 52.12 | 34.20 | 34.15 |
|  | Hemicellulose | 16.95 | 50.45 | 24.45 | 25.10 |
|  | Lignin | 0.77 | 9.77 | 5.54 | 5.46 |
| Biomass | Days to Flowering | 54 | 107 | 75 | 76.79 |
|  | Plant Height | 0.70 | 4.97 | 1.98 | 2.16 |
|  | FMY | 12.33 | 140.82 | 46.31 | 51.61 |
|  | DMY | 2.74 | 52.39 | 12.92 | 14.13 |
|  | ADF | 23.54 | 58.58 | 43.85 | 43.58 |
|  | NDF | 37.44 | 85.43 | 70.53 | 70.05 |
|  | Cellulose | 19.76 | 50.33 | 37.42 | 37.31 |
|  | Hemicellulose | 20.44 | 55.31 | 26.39 | 26.84 |
|  | Lignin | 0.95 | 10.51 | 6.26 | 6.27 |
