# Supplementary material for: Genomic prediction applied to high-biomass sorghum for bioenergy production
Source: Mol Breed. 2018 Apr 10;38(4):49. doi: 10.1007/s11032-018-0802-5 (PMC5893689; doi:10.1007/s11032-018-0802-5)
Supplement: Supplementary file 4 — (DOCX 15 kb) [file 11032_2018_802_MOESM4_ESM.docx]

**Online Resource 4**

**Article Title:** Genomic prediction applied to high biomass sorghum for bioenergy production

**Journal:** Molecular Breeding

**Authors:** Amanda Avelar de Oliveira; Maria Marta Pastina; Vander Filipe de Souza; Rafael Augusto da Costa Parrella; Roberto Willians Noda; Maria Lúcia Ferreira Simeone; Robert Eugene Schaffert; Jurandir Vieira de Magalhães; Cynthia Maria Borges Damasceno; Gabriel Rodrigues Alves Margarido.

**Name, affiliation, and email of corresponding author:**

Gabriel Rodrigues Alves Margarido

Escola Superior de Agricultura Luiz de Queiroz, USP

Piracicaba, SP 13418-900, Brazil

e-mail: gramarga@usp.br

Cynthia Maria Borges Damasceno

Embrapa Milho e Sorgo

Sete Lagoas, MG 35701-970, Brazil

e-mail: [cynthia.damasceno@embrapa.br](mailto:cynthia.damasceno@embrapa.br)

**Supplementary Table 4** The variance-covariance (VCOV) structure models selected for the random effects, with respective values of the BIC criterion for the sub-panel I

| Trait | Replicate/Year | Geno/Year | Block/Replicate/Year |  | Plot/Block/Replicate/Year | BIC |
| --- | --- | --- | --- | --- | --- | --- |
| Plant Height | UNS | UNS | DIAG $\otimes$ ID |  | ID$\otimes$ID$\otimes$ID | 615.15 |
| Cellulose | UNS | CS_HET_ | DIAG$\otimes$ID |  | ID$\otimes$DIAG$\otimes$ID | 3515.48 |
| ADF | UNS | UNS | DIAG$\otimes$ID |  | ID$\otimes$DIAG$\otimes$ID | 3690.67 |
| NDF | UNS | CS_HET_ | DIAG$\otimes$ID |  | DIAG$\otimes$ID$\otimes$ID | 3957.01 |
| Days to Flowering | DIAG | UNS | DIAG$\otimes$ID |  | DIAG$\otimes$ID$\otimes$ID | 3723.74 |
| Hemicellulose | DIAG | CS_HET_ | DIAG$\otimes$ID |  | DIAG$\otimes$DIAG$\otimes$DIAG | 3229.67 |
| Lignin | ID | CS_HET_ | DIAG$\otimes$ID |  | ID$\otimes$DIAG$\otimes$ID | 2053.75 |
| DMY | UNS | UNS | DIAG$\otimes$ID |  | DIAG$\otimes$DIAG$\otimes$ID | 3422.14 |
| FMY | DIAG | UNS | DIAG$\otimes$ID |  | DIAG$\otimes$ID$\otimes$ID | 4601.39 |

*ID* identity matrix, *DIAG* diagonal *CS_HET_* heterogeneous compound symmetry and *UNS* unstructured. The block effect models in the third column use the direct product ($\otimes)$between two component (co)variance matrices for replicate and year. The plot effect models in the fourth column use the direct product between tree component (co)variance matrices for block, replicate and year
