# Supplementary material for: Genomic prediction applied to high-biomass sorghum for bioenergy production
Source: Mol Breed. 2018 Apr 10;38(4):49. doi: 10.1007/s11032-018-0802-5 (PMC5893689; doi:10.1007/s11032-018-0802-5)
Supplement: Supplementary file 5 — (DOCX 14 kb) [file 11032_2018_802_MOESM5_ESM.docx]

**Online Resource 5**

**Article Title:** Genomic prediction applied to high biomass sorghum for bioenergy production

**Journal:** Molecular Breeding

**Authors:** Amanda Avelar de Oliveira; Maria Marta Pastina; Vander Filipe de Souza; Rafael Augusto da Costa Parrella; Roberto Willians Noda; Maria Lúcia Ferreira Simeone; Robert Eugene Schaffert; Jurandir Vieira de Magalhães; Cynthia Maria Borges Damasceno; Gabriel Rodrigues Alves Margarido.

**Name, affiliation, and email of corresponding author:**

Gabriel Rodrigues Alves Margarido

Escola Superior de Agricultura Luiz de Queiroz, USP

Piracicaba, SP 13418-900, Brazil

e-mail: gramarga@usp.br

Cynthia Maria Borges Damasceno

Embrapa Milho e Sorgo

Sete Lagoas, MG 35701-970, Brazil

e-mail: [cynthia.damasceno@embrapa.br](mailto:cynthia.damasceno@embrapa.br)

**Supplementary Table 5** The variance-covariance (VCOV) structure models selected for the random effects, with respective values of the BIC criterion for the sub-panel II

| Trait | Block/Replicate |  | Plot/Block/Replicate | BIC |
| --- | --- | --- | --- | --- |
| Plant Height | ID |  | ID$\otimes$ID | 367.30 |
| Cellulose | ID |  | ID$\otimes$ID | 1536.43 |
| ADF | ID |  | ID$\otimes$ID | 1723.11 |
| NDF | ID |  | ID$\otimes$ID | 1754.76 |
| Days to Flowering | ID |  | DIAG$\otimes$ID | 2116.85 |
| Hemicellulose | ID |  | ID$\otimes$ID | 1351.72 |
| Lignin | ID |  | ID$\otimes$ID | 1134.64 |
| DMY | ID |  | DIAG$\otimes$DIAG | 1381.00 |
| FMY | ID |  | DIAG$\otimes$ID | 1994.65 |

*ID* identity matrix, *DIAG* diagonal, *CS_HET_* heterogeneous compound symmetry and *UNS* unstructured. The block effect models in the second column use the ID. The plot effect models in the third column use the direct product ($\otimes$) between two component (co)variance matrices for block and replicate
