# Supplementary material for: Genomic prediction applied to high-biomass sorghum for bioenergy production
Source: Mol Breed. 2018 Apr 10;38(4):49. doi: 10.1007/s11032-018-0802-5 (PMC5893689; doi:10.1007/s11032-018-0802-5)
Supplement: Supplementary file 6 — (DOCX 17 kb) [file 11032_2018_802_MOESM6_ESM.docx]

**Online Resource 6**

**Article Title:** Genomic prediction applied to high biomass sorghum for bioenergy production

**Journal:** Molecular Breeding

**Authors:** Amanda Avelar de Oliveira; Maria Marta Pastina; Vander Filipe de Souza; Rafael Augusto da Costa Parrella; Roberto Willians Noda; Maria Lúcia Ferreira Simeone; Robert Eugene Schaffert; Jurandir Vieira de Magalhães; Cynthia Maria Borges Damasceno; Gabriel Rodrigues Alves Margarido.

**Name, affiliation, and email of corresponding author:**

Gabriel Rodrigues Alves Margarido

Escola Superior de Agricultura Luiz de Queiroz, USP

Piracicaba, SP 13418-900, Brazil

e-mail: gramarga@usp.br

Cynthia Maria Borges Damasceno

Embrapa Milho e Sorgo

Sete Lagoas, MG 35701-970, Brazil

e-mail: [cynthia.damasceno@embrapa.br](mailto:cynthia.damasceno@embrapa.br)

**Supplementary Table 6** Predictive abilities obtained in the validation within each sub-panel from six genomic selection models applied to nine traits of the high biomass sorghum panel of Embrapa Maize and Sorghum. Values indicate the correlation coefficient between the breeding values predicted by genomic selection models and the phenotypic breeding values

| Sub-Panel | Trait | Genomic Selection Model | | | | | |
| --- | --- | --- | --- | --- | --- | --- | --- |
|  |  | BayesB | BayesA | BayesRR | BayesC | BayesLasso | RRBLUP |
| I | Plant Height | 0.79 | 0.78 | 0.78 | 0.78 | 0.77 | 0.79 |
|  | Cellulose | 0.58 | 0.58 | 0.58 | 0.58 | 0.58 | 0.57 |
|  | ADF | 0.69 | 0.70 | 0.69 | 0.70 | 0.67 | 0.71 |
|  | NDF | 0.74 | 0.74 | 0.74 | 0.74 | 0.72 | 0.75 |
|  | Days to Flowering | 0.69 | 0.70 | 0.69 | 0.69 | 0.68 | 0.71 |
|  | Hemicellulose | 0.58 | 0.58 | 0.58 | 0.58 | 0.58 | 0.57 |
|  | Lignin | 0.44 | 0.44 | 0.44 | 0.44 | 0.44 | 0.43 |
|  | DMY | 0.73 | 0.73 | 0.73 | 0.73 | 0.71 | 0.74 |
|  | FMY | 0.60 | 0.60 | 0.59 | 0.60 | 0.57 | 0.61 |
| II | Plant Height | 0.80 | 0.80 | 0.80 | 0.80 | 0.79 | 0.81 |
|  | Cellulose | 0.65 | 0.65 | 0.65 | 0.65 | 0.65 | 0.65 |
|  | ADF | 0.65 | 0.65 | 0.65 | 0.65 | 0.65 | 0.65 |
|  | NDF | 0.59 | 0.59 | 0.59 | 0.59 | 0.59 | 0.59 |
|  | Days to Flowering | 0.45 | 0.46 | 0.45 | 0.45 | 0.39 | 0.49 |
|  | Hemicellulose | 0.16 | 0.16 | 0.14 | 0.15 | 0.10 | 0.13 |
|  | Lignin | 0.50 | 0.50 | 0.51 | 0.51 | 0.52 | 0.49 |
|  | DMY | 0.56 | 0.55 | 0.56 | 0.56 | 0.55 | 0.56 |
|  | FMY | 0.63 | 0.62 | 0.62 | 0.62 | 0.61 | 0.65 |
