# Supplementary material for: Genomic prediction applied to high-biomass sorghum for bioenergy production
Source: Mol Breed. 2018 Apr 10;38(4):49. doi: 10.1007/s11032-018-0802-5 (PMC5893689; doi:10.1007/s11032-018-0802-5)
Supplement: Supplementary file 7 — (DOCX 21 kb) [file 11032_2018_802_MOESM7_ESM.docx]

**Online Resource 7**

**Article Title:** Genomic prediction applied to high biomass sorghum for bioenergy production

**Journal:** Molecular Breeding

**Authors:** Amanda Avelar de Oliveira; Maria Marta Pastina; Vander Filipe de Souza; Rafael Augusto da Costa Parrella; Roberto Willians Noda; Maria Lúcia Ferreira Simeone; Robert Eugene Schaffert; Jurandir Vieira de Magalhães; Cynthia Maria Borges Damasceno; Gabriel Rodrigues Alves Margarido.

**Name, affiliation, and email of corresponding author:**

Gabriel Rodrigues Alves Margarido

Escola Superior de Agricultura Luiz de Queiroz, USP

Piracicaba, SP 13418-900, Brazil

e-mail: gramarga@usp.br

Cynthia Maria Borges Damasceno

Embrapa Milho e Sorgo

Sete Lagoas, MG 35701-970, Brazil

e-mail: [cynthia.damasceno@embrapa.br](mailto:cynthia.damasceno@embrapa.br)

**Supplementary Table 7** Predictive abilities obtained in the analyses across sub-panels from six genomic selection models applied to nine traits of the high biomass sorghum panel of Embrapa Maize and Sorghum. Values indicate the correlation coefficient between the breeding values predicted by genomic selection models and the phenotypic breeding values

|  | Heritability (training population) | Genomic Selection Model | | | | | |
| --- | --- | --- | --- | --- | --- | --- | --- |
|  |  | BayesB | BayesA | BayesRR | BayesC | BayesLasso | RRBLUP |
| Plant Height | 0.96 | 0.65 | 0.64 | 0.65 | 0.65 | 0.64 | 0.64 |
| Cellulose | 0.78 | 0.26 | 0.21 | 0.28 | 0.27 | 0.25 | 0.17 |
| ADF | 0.83 | 0.25 | 0.29 | 0.31 | 0.29 | 0.39 | 0.23 |
| NDF | 0.76 | 0.26 | 0.25 | 0.22 | 0.25 | 0.26 | 0.16 |
| Days to Flowering | 0.81 | 0.20 | 0.21 | 0.20 | 0.20 | 0.21 | 0.20 |
| Hemicellulose | 0.39 | -0.06 | -0.04 | -0.03 | -0.04 | -0.03 | -0.04 |
| Lignin | 0.82 | 0.36 | 0.34 | 0.36 | 0.35 | 0.38 | 0.34 |
| DMY | 0.70 | 0.33 | 0.33 | 0.33 | 0.33 | 0.35 | 0.33 |
| FMY | 0.80 | 0.25 | 0.25 | 0.25 | 0.24 | 0.26 | 0.25 |

**Supplementary Table 8** Predictive abilities obtained in the portability across subpanel, i.e., training in sub-panel I, year 1 and testing in sub-panel II, year 1, from six genomic selection models applied to nine traits of the high biomass sorghum panel of Embrapa Maize and Sorghum. Values indicate the correlation coefficient between the breeding values predicted by genomic selection models and the phenotypic breeding values

|  | Genomic Selection Model | | | | | |
| --- | --- | --- | --- | --- | --- | --- |
|  | BayesB | BayesA | BayesRR | BayesC | BayesLasso | RRBLUP |
| Plant Height | 0.66 | 0.65 | 0.65 | 0.66 | 0.65 | 0.65 |
| Cellulose | 0.41 | 0.43 | 0.40 | 0.39 | 0.43 | 0.36 |
| ADF | 0.42 | 0.43 | 0.41 | 0.41 | 0.44 | 0.38 |
| NDF | 0.27 | 0.31 | 0.28 | 0.27 | 0.31 | 0.22 |
| Days to Flowering | 0.24 | 0.24 | 0.25 | 0.27 | 0.25 | 0.25 |
| Hemicellulose | -0.05 | -0.01 | -0.03 | -0.04 | -0.01 | -0.04 |
| Lignin | 0.30 | 0.32 | 0.30 | 0.31 | 0.31 | 0.28 |
| DMY | 0.34 | 0.31 | 0.32 | 0.34 | 0.33 | 0.32 |
| FMY | 0.28 | 0.26 | 0.27 | 0.29 | 0.28 | 0.27 |

**Supplementary Table 9** Predictive abilities obtained in portability across sub-panels and years, i.e.; training in sub-panel I, year 2 and testing in sub-panel II, year 1, from six genomic selection models applied to nine traits of the high biomass sorghum panel of Embrapa Maize and Sorghum. Values indicate the correlation coefficient between the breeding values predicted by genomic selection models and the phenotypic breeding values

|  | Genomic Selection Model | | | | | |
| --- | --- | --- | --- | --- | --- | --- |
|  | BayesB | BayesA | BayesRR | BayesC | BayesLasso | RRBLUP |
| Plant Height | 0.63 | 0.63 | 0.64 | 0.63 | 0.64 | 0.64 |
| Cellulose | 0.05 | 0.02 | 0.05 | 0.02 | 0.13 | -0.05 |
| ADF | 0.09 | 0.14 | 0.12 | 0.14 | 0.25 | 0.04 |
| NDF | 0.18 | 0.18 | 0.19 | 0.16 | 0.23 | 0.11 |
| Days to Flowering | 0.21 | 0.21 | 0.21 | 0.21 | 0.21 | 0.21 |
| Hemicellulose | 0.003 | -0.03 | -0.004 | -0.06 | 0.05 | -0.02 |
| Lignin | 0.34 | 0.34 | 0.31 | 0.32 | 0.34 | 0.33 |
| DMY | 0.36 | 0.34 | 0.35 | 0.36 | 0.36 | 0.35 |
| FMY | 0.24 | 0.22 | 0.22 | 0.24 | 0.24 | 0.22 |

**Supplementary Table 10** Predictive abilities obtained in portability across years, i.e.; training in sub-panel I, year 1 and testing in sub-panel, I year 2, from six genomic selection models applied to nine traits of the high biomass sorghum panel of Embrapa Maize and Sorghum. Values indicate the correlation coefficient between the breeding values predicted by genomic selection models and the phenotypic breeding values

|  | Genomic Selection Model | | | | | |
| --- | --- | --- | --- | --- | --- | --- |
|  | BayesB | BayesA | BayesRR | BayesC | BayesLasso | RRBLUP |
| Plant Height | 0.95 | 0.95 | 0.95 | 0.95 | 0.95 | 0.98 |
| Cellulose | 0.72 | 0.71 | 0.72 | 0.72 | 0.74 | 0.66 |
| ADF | 0.76 | 0.75 | 0.76 | 0.75 | 0.77 | 0.71 |
| NDF | 0.68 | 0.69 | 0.69 | 0.69 | 0.71 | 0.64 |
| Days to Flowering | 0.78 | 0.79 | 0.79 | 0.79 | 0.79 | 0.76 |
| Hemicellulose | 0.50 | 0.50 | 0.50 | 0.51 | 0.50 | 0.49 |
| Lignin | 0.73 | 0.71 | 0.73 | 0.73 | 0.73 | 0.70 |
| DMY | 0.75 | 0.74 | 0.76 | 0.75 | 0.79 | 0.69 |
| FMY | 0.81 | 0.80 | 0.81 | 0.80 | 0.81 | 0.77 |
