# Supplementary material for: Genomic prediction applied to high-biomass sorghum for bioenergy production
Source: Mol Breed. 2018 Apr 10;38(4):49. doi: 10.1007/s11032-018-0802-5 (PMC5893689; doi:10.1007/s11032-018-0802-5)
Supplement: Supplementary file 8 — (DOCX 21 kb) [file 11032_2018_802_MOESM8_ESM.docx]

**Online Resource 8**

**Article Title:** Genomic prediction applied to high biomass sorghum for bioenergy production

**Journal:** Molecular Breeding

**Authors:** Amanda Avelar de Oliveira; Maria Marta Pastina; Vander Filipe de Souza; Rafael Augusto da Costa Parrella; Roberto Willians Noda; Maria Lúcia Ferreira Simeone; Robert Eugene Schaffert; Jurandir Vieira de Magalhães; Cynthia Maria Borges Damasceno; Gabriel Rodrigues Alves Margarido.

**Name, affiliation, and email of corresponding author:**

Gabriel Rodrigues Alves Margarido

Escola Superior de Agricultura Luiz de Queiroz, USP

Piracicaba, SP 13418-900, Brazil

e-mail: gramarga@usp.br

Cynthia Maria Borges Damasceno

Embrapa Milho e Sorgo

Sete Lagoas, MG 35701-970, Brazil

e-mail: [cynthia.damasceno@embrapa.br](mailto:cynthia.damasceno@embrapa.br)

**Supplementary Table 11** Results of the functional enrichment Kolmogorov-Smirnov test for the trait plant height. The false discovery rate corrected $p$-value and description for each enriched gene ontology term are shown.

| **GO term** | **- log_10_ p-value** | **Description** | **Number of markers** |
| --- | --- | --- | --- |
| GO:0006914 | 14.03 | autophagy | 101 |
| GO:0006099 | 11.93 | tricarboxylic acid cycle | 74 |
| GO:0016829 | 8.27 | lyase activity | 343 |
| GO:0046912 | 7.48 | transferase activity, transferring acyl groups, acyl groups converted into alkyl on transfer | 44 |
| GO:0044262 | 7.48 | cellular carbohydrate metabolic process | 44 |
| GO:0035299 | 7.48 | inositol pentakisphosphate 2-kinase activity | 12 |
| GO:0006633 | 7.38 | fatty acid biosynthetic process | 633 |
| GO:0003724 | 7.38 | RNA helicase activity | 25 |
| GO:0004146 | 7.38 | dihydrofolate reductase activity | 14 |
| GO:0006545 | 7.38 | glycine biosynthetic process | 14 |
| GO:0009165 | 7.38 | nucleotide biosynthetic process | 14 |
| GO:0004799 | 7.38 | thymidylate synthase activity | 14 |
| GO:0006231 | 7.38 | dTMP biosynthetic process | 14 |
| GO:0019748 | 7.38 | secondary metabolic process | 18 |
| GO:0008508 | 6.84 | bile acid:sodium symporter activity | 48 |
| GO:0005509 | 5.65 | calcium ion binding | 673 |
| GO:0006468 | 5.22 | protein phosphorylation | 9161 |
| GO:0016300 | 5.22 | tRNA (uracil) methyltransferase activity | 24 |
| GO:0002098 | 5.22 | tRNA wobble uridine modification | 24 |
| GO:0005525 | 5.21 | GTP binding | 919 |
| GO:0016998 | 5.05 | cell wall macromolecule catabolic process | 176 |
| GO:0007264 | 4.99 | small GTPase mediated signal transduction | 329 |
| GO:0004672 | 4.94 | protein kinase activity | 9137 |
| GO:0008375 | 4.84 | acetylglucosaminyltransferase activity | 239 |
| GO:0031072 | 4.80 | heat shock protein binding | 518 |
| GO:0004315 | 4.80 | 3-oxoacyl-[acyl-carrier-protein] synthase activity | 184 |
| GO:0051082 | 4.59 | unfolded protein binding | 166 |
| GO:0003913 | 4.21 | DNA photolyase activity | 63 |
| GO:0005247 | 4.15 | voltage-gated chloride channel activity | 95 |
| GO:0006821 | 4.15 | chloride transport | 95 |
| GO:0006511 | 4.08 | ubiquitin-dependent protein catabolic process | 509 |
| GO:0003677 | 3.97 | DNA binding | 6057 |
| GO:0003924 | 3.97 | GTPase activity | 541 |
| GO:0005506 | 3.86 | iron ion binding | 2567 |
| GO:0016987 | 3.86 | sigma factor activity | 66 |
| GO:0015035 | 3.85 | protein disulfide oxidoreductase activity | 249 |
| GO:0015991 | 3.84 | ATP hydrolysis coupled proton transport | 109 |
| GO:0055114 | 3.78 | oxidation-reduction process | 8245 |
| GO:0045454 | 3.73 | cell redox homeostasis | 507 |
| GO:0009055 | 3.62 | electron carrier activity | 3340 |
| GO:0004222 | 3.62 | metalloendopeptidase activity | 183 |
| GO:0006807 | 3.62 | nitrogen compound metabolic process | 111 |
| GO:0006450 | 3.54 | regulation of translational fidelity | 11 |
| GO:0043161 | 3.46 | proteasome-mediated ubiquitin-dependent protein catabolic process | 17 |
| GO:0005992 | 3.42 | trehalose biosynthetic process | 135 |
| GO:0016788 | 3.40 | hydrolase activity, acting on ester bonds | 1105 |
| GO:0004129 | 3.06 | cytochrome-c oxidase activity | 36 |
| GO:0008081 | 2.97 | phosphoric diester hydrolase activity | 85 |
| GO:0042254 | 2.94 | ribosome biogenesis | 63 |
| GO:0015144 | 2.94 | carbohydrate transmembrane transporter activity | 43 |
| GO:0034219 | 2.94 | carbohydrate transmembrane transport | 43 |
| GO:0046872 | 2.92 | metal ion binding | 1649 |
| GO:0015238 | 2.92 | drug transmembrane transporter activity | 492 |
| GO:0015297 | 2.92 | antiporter activity | 492 |
| GO:0006855 | 2.92 | drug transmembrane transport | 492 |
| GO:0035556 | 2.92 | intracellular signal transduction | 120 |
| GO:0046961 | 2.89 | proton-transporting ATPase activity, rotational mechanism | 91 |
| GO:0016884 | 2.89 | carbon-nitrogen ligase activity, with glutamine as amido-N-donor | 230 |
| GO:0020037 | 2.83 | heme binding | 3066 |
| GO:0008652 | 2.74 | cellular amino acid biosynthetic process | 154 |
| GO:0006814 | 2.74 | sodium ion transport | 112 |
| GO:0000774 | 2.74 | adenyl-nucleotide exchange factor activity | 41 |
| GO:0042803 | 2.74 | protein homodimerization activity | 41 |
| GO:0030515 | 2.74 | snoRNA binding | 16 |
| GO:0031120 | 2.74 | snRNA pseudouridine synthesis | 16 |
| GO:0004602 | 2.72 | glutathione peroxidase activity | 10 |
| GO:0048280 | 2.72 | vesicle fusion with Golgi apparatus | 6 |
| GO:0004563 | 2.70 | beta-N-acetylhexosaminidase activity | 34 |
| GO:0005739 | 2.64 | mitochondrion | 24 |
| GO:0006457 | 2.60 | protein folding | 525 |
| GO:0016810 | 2.60 | hydrolase activity, acting on carbon-nitrogen (but not peptide) bonds | 69 |
| GO:0005337 | 2.60 | nucleoside transmembrane transporter activity | 19 |
| GO:0033178 | 2.55 | proton-transporting two-sector ATPase complex, catalytic domain | 75 |
| GO:0006952 | 2.55 | defense response | 124 |
| GO:0030337 | 2.55 | DNA polymerase processivity factor activity | 25 |
| GO:0006275 | 2.55 | regulation of DNA replication | 25 |
| GO:0043626 | 2.55 | PCNA complex | 25 |
| GO:0031227 | 2.53 | intrinsic component of endoplasmic reticulum membrane | 91 |
| GO:0016020 | 2.45 | membrane | 6374 |
| GO:0004553 | 2.39 | hydrolase activity, hydrolyzing O-glycosyl compounds | 1849 |
| GO:0005975 | 2.37 | carbohydrate metabolic process | 2696 |
| GO:0008374 | 2.32 | O-acyltransferase activity | 94 |
| GO:0005524 | 2.29 | ATP binding | 14385 |
| GO:0043531 | 2.29 | ADP binding | 2507 |
| GO:0008270 | 2.26 | zinc ion binding | 3663 |
| GO:0006505 | 2.23 | GPI anchor metabolic process | 61 |
| GO:0005737 | 2.23 | cytoplasm | 1266 |
| GO:0004650 | 2.23 | polygalacturonase activity | 318 |
| GO:0004832 | 2.17 | valine-tRNA ligase activity | 12 |
| GO:0006438 | 2.17 | valyl-tRNA aminoacylation | 12 |
| GO:0050080 | 2.15 | malonyl-CoA decarboxylase activity | 13 |
| GO:0010333 | 2.13 | terpene synthase activity | 153 |
| GO:0016844 | 2.12 | strictosidine synthase activity | 105 |
| GO:0017038 | 2.07 | protein import | 42 |
