# Supplementary material for: Genomic prediction applied to high-biomass sorghum for bioenergy production
Source: Mol Breed. 2018 Apr 10;38(4):49. doi: 10.1007/s11032-018-0802-5 (PMC5893689; doi:10.1007/s11032-018-0802-5)
Supplement: Supplementary file 9 — (DOCX 19 kb) [file 11032_2018_802_MOESM9_ESM.docx]

**Online Resource 9**

**Article Title:** Genomic prediction applied to high biomass sorghum for bioenergy production

**Journal:** Molecular Breeding

**Authors:** Amanda Avelar de Oliveira; Maria Marta Pastina; Vander Filipe de Souza; Rafael Augusto da Costa Parrella; Roberto Willians Noda; Maria Lúcia Ferreira Simeone; Robert Eugene Schaffert; Jurandir Vieira de Magalhães; Cynthia Maria Borges Damasceno; Gabriel Rodrigues Alves Margarido.

**Name, affiliation, and email of corresponding author:**

Gabriel Rodrigues Alves Margarido

Escola Superior de Agricultura Luiz de Queiroz, USP

Piracicaba, SP 13418-900, Brazil

e-mail: gramarga@usp.br

Cynthia Maria Borges Damasceno

Embrapa Milho e Sorgo

Sete Lagoas, MG 35701-970, Brazil

e-mail: [cynthia.damasceno@embrapa.br](mailto:cynthia.damasceno@embrapa.br)

**Supplementary Table 12** Results of the functional enrichment Kolmogorov-Smirnov test for the trait cellulose. The false discovery rate corrected $p$-value and description for each enriched gene ontology term are shown.

| **GO term** | **-log_10_ p-value** | **Description** | **Number of markers** |
| --- | --- | --- | --- |
| GO:0003871 | 11.68 | 5-methyltetrahydropteroyltriglutamate-homocysteine S-methyltransferase activity | 55 |
| GO:0009086 | 11.68 | methionine biosynthetic process | 55 |
| GO:0006164 | 10.76 | purine nucleotide biosynthetic process | 41 |
| GO:0008759 | 10.76 | UDP-3-O-[3-hydroxymyristoyl] N-acetylglucosamine deacetylase activity | 16 |
| GO:0005351 | 10.29 | Sugar proton symporter activity | 52 |
| GO:0008643 | 10.29 | carbohydrate transport | 52 |
| GO:0003937 | 10.29 | IMP cyclohydrolase activity | 36 |
| GO:0004643 | 10.29 | phosphoribosylaminoimidazolecarboxamide formyltransferase activity | 36 |
| GO:0008276 | 9.83 | protein methyltransferase activity | 139 |
| GO:0006479 | 9.83 | protein methylation | 139 |
| GO:0006505 | 8.10 | GPI anchor metabolic process | 61 |
| GO:0006629 | 7.98 | lipid metabolic process | 1739 |
| GO:0008236 | 7.54 | serine-type peptidase activity | 266 |
| GO:0019748 | 7.38 | secondary metabolic process | 18 |
| GO:0016788 | 7.37 | hydrolase activity, acting on ester bonds | 1105 |
| GO:0000139 | 7.35 | Golgi membrane | 61 |
| GO:0004806 | 6.67 | triglyceride lipase activity | 379 |
| GO:0016300 | 6.25 | tRNA (uracil) methyltransferase activity | 24 |
| GO:0002098 | 6.25 | tRNA wobble uridine modification | 24 |
| GO:0009245 | 6.24 | lipid A biosynthetic process | 26 |
| GO:0043531 | 6.22 | ADP binding | 2507 |
| GO:0004146 | 6.22 | dihydrofolate reductase activity | 14 |
| GO:0006545 | 6.22 | glycine biosynthetic process | 14 |
| GO:0009165 | 6.22 | nucleotide biosynthetic process | 14 |
| GO:0004799 | 6.22 | thymidylate synthase activity | 14 |
| GO:0006231 | 6.22 | dTMP biosynthetic process | 14 |
| GO:0004176 | 6.20 | ATP-dependent peptidase activity | 103 |
| GO:0015105 | 6.15 | arsenite transmembrane transporter activity | 55 |
| GO:0004003 | 6.10 | ATP-dependent DNA helicase activity | 56 |
| GO:0006450 | 4.94 | regulation of translational fidelity | 11 |
| GO:0000042 | 4.82 | protein targeting to Golgi | 18 |
| GO:0009306 | 4.75 | protein secretion | 18 |
| GO:0007018 | 4.57 | microtubule-based movement | 288 |
| GO:0031227 | 4.55 | intrinsic component of endoplasmic reticulum membrane | 91 |
| GO:0010333 | 4.00 | terpene synthase activity | 153 |
| GO:0017038 | 3.85 | protein import | 42 |
| GO:0005788 | 3.58 | endoplasmic reticulum lumen | 24 |
| GO:0008508 | 3.56 | bile acid:sodium symporter activity | 48 |
| GO:0043169 | 3.44 | cation binding | 127 |
| GO:0050790 | 3.39 | regulation of catalytic activity | 9 |
| GO:0008652 | 3.38 | cellular amino acid biosynthetic process | 154 |
| GO:0051258 | 3.38 | protein polymerization | 40 |
| GO:0043234 | 3.38 | protein complex | 40 |
| GO:0006568 | 3.20 | tryptophan metabolic process | 14 |
| GO:0016020 | 2.97 | membrane | 6374 |
| GO:0003735 | 2.97 | structural constituent of ribosome | 782 |
| GO:0005840 | 2.97 | ribosome | 752 |
| GO:0046983 | 2.97 | protein dimerization activity | 1135 |
| GO:0006184 | 2.96 | obsolete GTP catabolic process | 43 |
| GO:0045132 | 2.84 | meiotic chromosome segregation | 34 |
| GO:0015137 | 2.81 | citrate transmembrane transporter activity | 107 |
| GO:0015746 | 2.81 | citrate transport | 107 |
| GO:0004834 | 2.80 | tryptophan synthase activity | 13 |
| GO:0004674 | 2.77 | protein serine/threonine kinase activity | 82 |
| GO:0006414 | 2.63 | translational elongation | 61 |
| GO:0004553 | 2.47 | hydrolase activity, hydrolyzing O-glycosyl compounds | 1849 |
| GO:0016798 | 2.43 | hydrolase activity, acting on glycosyl bonds | 64 |
| GO:0005086 | 2.42 | ARF guanyl-nucleotide exchange factor activity | 38 |
| GO:0032012 | 2.42 | regulation of ARF protein signal transduction | 38 |
| GO:0009439 | 2.38 | cyanate metabolic process | 8 |
| GO:0016458 | 2.38 | gene silencing | 12 |
| GO:0008963 | 2.31 | phospho-N-acetylmuramoyl-pentapeptide-transferase activity | 18 |
| GO:0004519 | 2.24 | endonuclease activity | 75 |
| GO:0004784 | 2.23 | superoxide dismutase activity | 20 |
| GO:0003913 | 2.19 | DNA photolyase activity | 63 |
| GO:0016070 | 2.14 | RNA metabolic process | 50 |
| GO:0015078 | 2.11 | hydrogen ion transmembrane transporter activity | 30 |
| GO:0016706 | 2.09 | oxidoreductase activity, acting on paired donors, with incorporation or reduction of molecular oxygen, 2-oxoglutarate as one donor, and incorporation of one atom each of oxygen into both donors | 591 |
| GO:0015238 | 2.06 | drug transmembrane transporter activity | 492 |
| GO:0015297 | 2.06 | antiporter activity | 492 |
| GO:0006855 | 2.06 | drug transmembrane transport | 492 |
| GO:0016772 | 2.03 | transferase activity, transferring phosphorus-containing groups | 198 |
| GO:0004970 | 2.02 | ionotropic glutamate receptor activity | 205 |
| GO:0005234 | 2.02 | extracellular-glutamate-gated ion channel activity | 205 |
