# Supplementary material for: Genomic prediction applied to high-biomass sorghum for bioenergy production
Source: Mol Breed. 2018 Apr 10;38(4):49. doi: 10.1007/s11032-018-0802-5 (PMC5893689; doi:10.1007/s11032-018-0802-5)
Supplement: Supplementary file 10 — (DOCX 20 kb) [file 11032_2018_802_MOESM10_ESM.docx]

**Online Resource 10**

**Article Title:** Genomic prediction applied to high biomass sorghum for bioenergy production

**Journal:** Molecular Breeding

**Authors:** Amanda Avelar de Oliveira; Maria Marta Pastina; Vander Filipe de Souza; Rafael Augusto da Costa Parrella; Roberto Willians Noda; Maria Lúcia Ferreira Simeone; Robert Eugene Schaffert; Jurandir Vieira de Magalhães; Cynthia Maria Borges Damasceno; Gabriel Rodrigues Alves Margarido.

**Name, affiliation, and email of corresponding author:**

Gabriel Rodrigues Alves Margarido

Escola Superior de Agricultura Luiz de Queiroz, USP

Piracicaba, SP 13418-900, Brazil

e-mail: gramarga@usp.br

Cynthia Maria Borges Damasceno

Embrapa Milho e Sorgo

Sete Lagoas, MG 35701-970, Brazil

e-mail: [cynthia.damasceno@embrapa.br](mailto:cynthia.damasceno@embrapa.br)

**Supplementary Table 13** Results of the functional enrichment Kolmogorov-Smirnov test for the trait acid detergent fiber. The false discovery rate corrected $p$-value and description for each enriched gene ontology term are shown.

| **GO term** | **- log_10_ p-value** | **Description** | **Number of markers** |
| --- | --- | --- | --- |
| GO:0008759 | 9.70 | UDP-3-O-[3-hydroxymyristoyl] N-acetylglucosamine deacetylase activity | 16 |
| GO:0006164 | 9.20 | purine nucleotide biosynthetic process | 41 |
| GO:0003871 | 9.20 | 5-methyltetrahydropteroyltriglutamate-homocysteine S-methyltransferase activity | 55 |
| GO:0009086 | 9.20 | methionine biosynthetic process | 55 |
| GO:0003937 | 8.81 | IMP cyclohydrolase activity | 36 |
| GO:0004643 | 8.81 | phosphoribosylaminoimidazolecarboxamide formyltransferase activity | 36 |
| GO:0006505 | 8.09 | GPI anchor metabolic process | 61 |
| GO:0000042 | 7.72 | protein targeting to Golgi | 18 |
| GO:0006914 | 7.72 | autophagy | 101 |
| GO:0004146 | 7.72 | dihydrofolate reductase activity | 14 |
| GO:0006545 | 7.72 | glycine biosynthetic process | 14 |
| GO:0009165 | 7.72 | nucleotide biosynthetic process | 14 |
| GO:0004799 | 7.72 | thymidylate synthase activity | 14 |
| GO:0006231 | 7.72 | dTMP biosynthetic process | 14 |
| GO:0008276 | 7.65 | protein methyltransferase activity | 139 |
| GO:0006479 | 7.65 | protein methylation | 139 |
| GO:0008508 | 7.60 | bile acid:sodium symporter activity | 48 |
| GO:0019748 | 7.35 | secondary metabolic process | 18 |
| GO:0004176 | 7.16 | ATP-dependent peptidase activity | 103 |
| GO:0008104 | 6.87 | protein localization | 94 |
| GO:0009245 | 6.69 | lipid A biosynthetic process | 26 |
| GO:0005351 | 6.41 | sugar:proton symporter activity | 52 |
| GO:0008643 | 6.41 | carbohydrate transport | 52 |
| GO:0016300 | 6.04 | tRNA (uracil) methyltransferase activity | 24 |
| GO:0002098 | 6.04 | tRNA wobble uridine modification | 24 |
| GO:0031227 | 5.65 | intrinsic component of endoplasmic reticulum membrane | 91 |
| GO:0015105 | 4.75 | arsenite transmembrane transporter activity | 55 |
| GO:0016458 | 4.62 | gene silencing | 12 |
| GO:0045132 | 4.49 | meiotic chromosome segregation | 34 |
| GO:0000139 | 4.49 | Golgi membrane | 61 |
| GO:0006450 | 4.43 | regulation of translational fidelity | 11 |
| GO:0004565 | 4.38 | beta-galactosidase activity | 92 |
| GO:0009341 | 4.38 | beta-galactosidase complex | 92 |
| GO:0051082 | 4.28 | unfolded protein binding | 166 |
| GO:0006857 | 4.10 | oligopeptide transport | 814 |
| GO:0004518 | 3.96 | nuclease activity | 107 |
| GO:0016070 | 3.89 | RNA metabolic process | 50 |
| GO:0016020 | 3.88 | membrane | 6374 |
| GO:0003777 | 3.79 | microtubule motor activity | 288 |
| GO:0007018 | 3.79 | microtubule-based movement | 288 |
| GO:0006457 | 3.66 | protein folding | 525 |
| GO:0003723 | 3.62 | RNA binding | 1179 |
| GO:0006568 | 3.61 | tryptophan metabolic process | 14 |
| GO:0003724 | 3.59 | RNA helicase activity | 25 |
| GO:0000774 | 3.47 | adenyl-nucleotide exchange factor activity | 41 |
| GO:0042803 | 3.47 | protein homodimerization activity | 41 |
| GO:0004834 | 3.29 | tryptophan synthase activity | 13 |
| GO:0004970 | 3.28 | ionotropic glutamate receptor activity | 205 |
| GO:0005234 | 3.28 | extracellular-glutamate-gated ion channel activity | 205 |
| GO:0043169 | 3.26 | cation binding | 127 |
| GO:0004003 | 3.25 | ATP-dependent DNA helicase activity | 56 |
| GO:0043531 | 3.25 | ADP binding | 2507 |
| GO:0010333 | 3.25 | terpene synthase activity | 153 |
| GO:0003951 | 3.23 | NAD+ kinase activity | 15 |
| GO:0030288 | 3.18 | outer membrane-bounded periplasmic space | 207 |
| GO:0008963 | 3.15 | phospho-N-acetylmuramoyl-pentapeptide-transferase activity | 18 |
| GO:0005198 | 2.97 | structural molecule activity | 197 |
| GO:0007275 | 2.97 | multicellular organismal development | 277 |
| GO:0005507 | 2.96 | copper ion binding | 502 |
| GO:0005215 | 2.93 | transporter activity | 1529 |
| GO:0016787 | 2.91 | hydrolase activity | 1764 |
| GO:0016310 | 2.85 | phosphorylation | 100 |
| GO:0006184 | 2.81 | obsolete GTP catabolic process | 43 |
| GO:0015137 | 2.81 | citrate transmembrane transporter activity | 107 |
| GO:0015746 | 2.81 | citrate transport | 107 |
| GO:0004348 | 2.78 | glucosylceramidase activity | 11 |
| GO:0006665 | 2.78 | sphingolipid metabolic process | 11 |
| GO:0019538 | 2.70 | protein metabolic process | 108 |
| GO:0001104 | 2.70 | RNA polymerase II transcription cofactor activity | 141 |
| GO:0006357 | 2.70 | regulation of transcription from RNA polymerase II promoter | 141 |
| GO:0016592 | 2.70 | mediator complex | 141 |
| GO:0016772 | 2.65 | transferase activity, transferring phosphorus-containing groups | 198 |
| GO:0004553 | 2.63 | hydrolase activity, hydrolyzing O-glycosyl compounds | 1849 |
| GO:0044237 | 2.55 | cellular metabolic process | 922 |
| GO:0000775 | 2.47 | chromosome, centromeric region | 51 |
| GO:0016788 | 2.44 | hydrolase activity, acting on ester bonds | 1105 |
| GO:0051258 | 2.40 | protein polymerization | 40 |
| GO:0043234 | 2.40 | protein complex | 40 |
| GO:0009306 | 2.40 | protein secretion | 18 |
| GO:0008324 | 2.37 | cation transmembrane transporter activity | 89 |
| GO:0008417 | 2.37 | fucosyltransferase activity | 9 |
| GO:0004842 | 2.36 | ubiquitin-protein transferase activity | 481 |
| GO:0008652 | 2.35 | cellular amino acid biosynthetic process | 154 |
| GO:0031072 | 2.35 | heat shock protein binding | 518 |
| GO:0016998 | 2.33 | cell wall macromolecule catabolic process | 176 |
| GO:0003697 | 2.33 | single-stranded DNA binding | 98 |
| GO:0004719 | 2.25 | protein-L-isoaspartate (D-aspartate) O-methyltransferase activity | 31 |
| GO:0006511 | 2.14 | ubiquitin-dependent protein catabolic process | 509 |
| GO:0006355 | 2.08 | regulation of transcription, DNA-templated | 6246 |
| GO:0016161 | 2.05 | beta-amylase activity | 120 |
| GO:0000272 | 2.05 | polysaccharide catabolic process | 120 |
| GO:0055085 | 2.04 | transmembrane transport | 4185 |
| GO:0008375 | 2.03 | acetylglucosaminyltransferase activity | 239 |
