# Supplementary material for: Genomic prediction applied to high-biomass sorghum for bioenergy production
Source: Mol Breed. 2018 Apr 10;38(4):49. doi: 10.1007/s11032-018-0802-5 (PMC5893689; doi:10.1007/s11032-018-0802-5)
Supplement: Supplementary file 11 — (DOCX 22 kb) [file 11032_2018_802_MOESM11_ESM.docx]

**Online Resource 11**

**Article Title:** Genomic prediction applied to high biomass sorghum for bioenergy production

**Journal:** Molecular Breeding

**Authors:** Amanda Avelar de Oliveira; Maria Marta Pastina; Vander Filipe de Souza; Rafael Augusto da Costa Parrella; Roberto Willians Noda; Maria Lúcia Ferreira Simeone; Robert Eugene Schaffert; Jurandir Vieira de Magalhães; Cynthia Maria Borges Damasceno; Gabriel Rodrigues Alves Margarido.

**Name, affiliation, and email of corresponding author:**

Gabriel Rodrigues Alves Margarido

Escola Superior de Agricultura Luiz de Queiroz, USP

Piracicaba, SP 13418-900, Brazil

e-mail: gramarga@usp.br

Cynthia Maria Borges Damasceno

Embrapa Milho e Sorgo

Sete Lagoas, MG 35701-970, Brazil

e-mail: [cynthia.damasceno@embrapa.br](mailto:cynthia.damasceno@embrapa.br)

**Supplementary Table 14** Results of the functional enrichment Kolmogorov-Smirnov test for the trait neutral detergent fiber. The false discovery rate corrected $p$-value and description for each enriched gene ontology term are shown.

| **GO term** | **- log_10_ p-value** | **Description** | **Number of markers** |
| --- | --- | --- | --- |
| GO:0004176 | 15.50 | ATP-dependent peptidase activity | 103 |
| GO:0008759 | 10.85 | UDP-3-O-[3-hydroxymyristoyl] N-acetylglucosamine deacetylase activity | 16 |
| GO:0016772 | 10.27 | transferase activity, transferring phosphorus-containing groups | 198 |
| GO:0004146 | 8.39 | dihydrofolate reductase activity | 14 |
| GO:0006545 | 8.39 | glycine biosynthetic process | 14 |
| GO:0009165 | 8.39 | nucleotide biosynthetic process | 14 |
| GO:0004799 | 8.39 | thymidylate synthase activity | 14 |
| GO:0006231 | 8.39 | dTMP biosynthetic process | 14 |
| GO:0008508 | 7.69 | bile acid:sodium symporter activity | 48 |
| GO:0019748 | 7.25 | secondary metabolic process | 18 |
| GO:0006694 | 7.19 | steroid biosynthetic process | 716 |
| GO:0010333 | 7.05 | terpene synthase activity | 153 |
| GO:0005351 | 7.05 | sugar:proton symporter activity | 52 |
| GO:0008643 | 7.05 | carbohydrate transport | 52 |
| GO:0016616 | 6.91 | oxidoreductase activity, acting on the CH-OH group of donors, NAD or NADP as acceptor | 990 |
| GO:0003854 | 6.71 | 3-beta-hydroxy-delta5-steroid dehydrogenase activity | 675 |
| GO:0031227 | 6.64 | intrinsic component of endoplasmic reticulum membrane | 91 |
| GO:0003871 | 6.64 | 5-methyltetrahydropteroyltriglutamate-homocysteine S-methyltransferase activity | 55 |
| GO:0009086 | 6.64 | methionine biosynthetic process | 55 |
| GO:0008276 | 6.64 | protein methyltransferase activity | 139 |
| GO:0006479 | 6.64 | protein methylation | 139 |
| GO:0006505 | 6.64 | GPI anchor metabolic process | 61 |
| GO:0009245 | 6.56 | lipid A biosynthetic process | 26 |
| GO:0035556 | 6.44 | intracellular signal transduction | 120 |
| GO:0000042 | 6.41 | protein targeting to Golgi | 18 |
| GO:0016300 | 5.95 | tRNA (uracil) methyltransferase activity | 24 |
| GO:0002098 | 5.95 | tRNA wobble uridine modification | 24 |
| GO:0009306 | 5.95 | protein secretion | 18 |
| GO:0005788 | 5.87 | endoplasmic reticulum lumen | 24 |
| GO:0015105 | 5.69 | arsenite transmembrane transporter activity | 55 |
| GO:0035299 | 5.46 | inositol pentakisphosphate 2-kinase activity | 12 |
| GO:0008104 | 5.18 | protein localization | 94 |
| GO:0044237 | 4.99 | cellular metabolic process | 922 |
| GO:0008652 | 4.98 | cellular amino acid biosynthetic process | 154 |
| GO:0006450 | 4.98 | regulation of translational fidelity | 11 |
| GO:0004003 | 4.88 | ATP-dependent DNA helicase activity | 56 |
| GO:0004553 | 4.84 | hydrolase activity, hydrolyzing O-glycosyl compounds | 1849 |
| GO:0008963 | 4.84 | phospho-N-acetylmuramoyl-pentapeptide-transferase activity | 18 |
| GO:0043531 | 4.80 | ADP binding | 2507 |
| GO:0043169 | 4.80 | cation binding | 127 |
| GO:0003937 | 4.80 | IMP cyclohydrolase activity | 36 |
| GO:0004643 | 4.80 | phosphoribosylaminoimidazolecarboxamide formyltransferase activity | 36 |
| GO:0005975 | 4.54 | carbohydrate metabolic process | 2696 |
| GO:0006568 | 4.54 | tryptophan metabolic process | 14 |
| GO:0051082 | 4.51 | unfolded protein binding | 166 |
| GO:0004143 | 4.44 | diacylglycerol kinase activity | 88 |
| GO:0007205 | 4.44 | protein kinase C-activating G-protein coupled receptor signaling pathway | 88 |
| GO:0031072 | 4.29 | heat shock protein binding | 518 |
| GO:0000139 | 4.27 | Golgi membrane | 61 |
| GO:0004834 | 4.17 | tryptophan synthase activity | 13 |
| GO:0004657 | 4.07 | proline dehydrogenase activity | 44 |
| GO:0006562 | 4.07 | proline catabolic process | 44 |
| GO:0017038 | 4.04 | protein import | 42 |
| GO:0033615 | 3.98 | mitochondrial proton-transporting ATP synthase complex assembly | 20 |
| GO:0015137 | 3.77 | citrate transmembrane transporter activity | 107 |
| GO:0015746 | 3.77 | citrate transport | 107 |
| GO:0003724 | 3.77 | RNA helicase activity | 25 |
| GO:0016310 | 3.74 | phosphorylation | 100 |
| GO:0050790 | 3.69 | regulation of catalytic activity | 9 |
| GO:0003951 | 3.51 | NAD+ kinase activity | 15 |
| GO:0006164 | 3.48 | purine nucleotide biosynthetic process | 41 |
| GO:0009439 | 3.47 | cyanate metabolic process | 8 |
| GO:0000105 | 3.34 | histidine biosynthetic process | 27 |
| GO:0003887 | 3.33 | DNA-directed DNA polymerase activity | 202 |
| GO:0015078 | 3.29 | hydrogen ion transmembrane transporter activity | 30 |
| GO:0004842 | 3.16 | ubiquitin-protein transferase activity | 481 |
| GO:0004519 | 3.09 | endonuclease activity | 75 |
| GO:0003913 | 3.01 | DNA photolyase activity | 63 |
| GO:0016620 | 3.00 | oxidoreductase activity, acting on the aldehyde or oxo group of donors, NAD or NADP as acceptor | 154 |
| GO:0006511 | 2.98 | ubiquitin-dependent protein catabolic process | 509 |
| GO:0015923 | 2.89 | mannosidase activity | 67 |
| GO:0006013 | 2.89 | mannose metabolic process | 67 |
| GO:0016567 | 2.89 | protein ubiquitination | 469 |
| GO:0000151 | 2.89 | ubiquitin ligase complex | 469 |
| GO:0004559 | 2.88 | alpha-mannosidase activity | 70 |
| GO:0005743 | 2.84 | mitochondrial inner membrane | 2 |
| GO:0045454 | 2.75 | cell redox homeostasis | 507 |
| GO:0007275 | 2.75 | multicellular organismal development | 277 |
| GO:0005247 | 2.57 | voltage-gated chloride channel activity | 95 |
| GO:0006821 | 2.57 | chloride transport | 95 |
| GO:0050662 | 2.56 | coenzyme binding | 1041 |
| GO:0015035 | 2.51 | protein disulfide oxidoreductase activity | 249 |
| GO:0004652 | 2.51 | polynucleotide adenylyltransferase activity | 59 |
| GO:0043631 | 2.51 | RNA polyadenylation | 59 |
| GO:0006468 | 2.47 | protein phosphorylation | 9161 |
| GO:0000287 | 2.40 | magnesium ion binding | 326 |
| GO:0016884 | 2.40 | carbon-nitrogen ligase activity, with glutamine as amido-N-donor | 230 |
| GO:0009058 | 2.38 | biosynthetic process | 1537 |
| GO:0008610 | 2.37 | lipid biosynthetic process | 449 |
| GO:0004970 | 2.36 | ionotropic glutamate receptor activity | 205 |
| GO:0005234 | 2.36 | extracellular-glutamate-gated ion channel activity | 205 |
| GO:0045300 | 2.35 | acyl-[acyl-carrier-protein] desaturase activity | 42 |
| GO:0004197 | 2.30 | cysteine-type endopeptidase activity | 158 |
| GO:0004672 | 2.25 | protein kinase activity | 9137 |
| GO:0019001 | 2.21 | guanyl nucleotide binding | 64 |
| GO:0007186 | 2.21 | G-protein coupled receptor signaling pathway | 64 |
| GO:0004806 | 2.17 | triglyceride lipase activity | 379 |
| GO:0016070 | 2.15 | RNA metabolic process | 50 |
| GO:0009378 | 2.11 | four-way junction helicase activity | 232 |
| GO:0030288 | 2.11 | outer membrane-bounded periplasmic space | 207 |
| GO:0004655 | 2.09 | porphobilinogen synthase activity | 25 |
| GO:0016021 | 2.08 | integral component of membrane | 4252 |
| GO:0030246 | 2.05 | carbohydrate binding | 60 |
| GO:0006508 | 2.05 | proteolysis | 2781 |
