# Supplementary material for: Genomic prediction applied to high-biomass sorghum for bioenergy production
Source: Mol Breed. 2018 Apr 10;38(4):49. doi: 10.1007/s11032-018-0802-5 (PMC5893689; doi:10.1007/s11032-018-0802-5)
Supplement: Supplementary file 12 — (DOCX 18 kb) [file 11032_2018_802_MOESM12_ESM.docx]

**Online Resource 12**

**Article Title:** Genomic prediction applied to high biomass sorghum for bioenergy production

**Journal:** Molecular Breeding

**Authors:** Amanda Avelar de Oliveira; Maria Marta Pastina; Vander Filipe de Souza; Rafael Augusto da Costa Parrella; Roberto Willians Noda; Maria Lúcia Ferreira Simeone; Robert Eugene Schaffert; Jurandir Vieira de Magalhães; Cynthia Maria Borges Damasceno; Gabriel Rodrigues Alves Margarido.

**Name, affiliation, and email of corresponding author:**

Gabriel Rodrigues Alves Margarido

Escola Superior de Agricultura Luiz de Queiroz, USP

Piracicaba, SP 13418-900, Brazil

e-mail: gramarga@usp.br

Cynthia Maria Borges Damasceno

Embrapa Milho e Sorgo

Sete Lagoas, MG 35701-970, Brazil

e-mail: [cynthia.damasceno@embrapa.br](mailto:cynthia.damasceno@embrapa.br)

**Supplementary Table 15** Results of the functional enrichment Kolmogorov-Smirnov test for the trait days to flowering. The false discovery rate corrected $p$-value and description for each enriched gene ontology term are shown.

| **GO term** | **- log_10_ p-value** | **Description** | **Number of markers** |
| --- | --- | --- | --- |
| GO:0006914 | 17.87 | autophagy | 101 |
| GO:0005509 | 10.23 | calcium ion binding | 673 |
| GO:0016788 | 10.08 | hydrolase activity, acting on ester bonds | 1105 |
| GO:0003937 | 8.68 | IMP cyclohydrolase activity | 36 |
| GO:0004643 | 8.68 | phosphoribosylaminoimidazolecarboxamide formyltransferase activity | 36 |
| GO:0004176 | 8.15 | ATP-dependent peptidase activity | 103 |
| GO:0043169 | 7.30 | cation binding | 127 |
| GO:0006164 | 7.11 | purine nucleotide biosynthetic process | 41 |
| GO:0019748 | 6.25 | secondary metabolic process | 18 |
| GO:0006099 | 6.05 | tricarboxylic acid cycle | 74 |
| GO:0016311 | 5.49 | dephosphorylation | 22 |
| GO:0000042 | 5.43 | protein targeting to Golgi | 18 |
| GO:0003871 | 5.37 | 5-methyltetrahydropteroyltriglutamate-homocysteine S-methyltransferase activity | 55 |
| GO:0009086 | 5.37 | methionine biosynthetic process | 55 |
| GO:0008026 | 4.62 | ATP-dependent helicase activity | 676 |
| GO:0016772 | 4.52 | transferase activity, transferring phosphorus-containing groups | 198 |
| GO:0051258 | 4.44 | protein polymerization | 40 |
| GO:0043234 | 4.44 | protein complex | 40 |
| GO:0003910 | 4.21 | DNA ligase (ATP) activity | 25 |
| GO:0003777 | 3.67 | microtubule motor activity | 288 |
| GO:0007018 | 3.67 | microtubule-based movement | 288 |
| GO:0004066 | 3.56 | asparagine synthase (glutamine-hydrolyzing) activity | 82 |
| GO:0006529 | 3.56 | asparagine biosynthetic process | 82 |
| GO:0016791 | 3.56 | phosphatase activity | 41 |
| GO:0006904 | 3.56 | vesicle docking involved in exocytosis | 300 |
| GO:0000226 | 3.56 | microtubule cytoskeleton organization | 53 |
| GO:0000922 | 3.56 | spindle pole | 53 |
| GO:0005815 | 3.56 | microtubule organizing center | 53 |
| GO:0006801 | 3.49 | superoxide metabolic process | 45 |
| GO:0016458 | 3.41 | gene silencing | 12 |
| GO:0006465 | 3.35 | signal peptide processing | 14 |
| GO:0005787 | 3.35 | signal peptidase complex | 14 |
| GO:0004129 | 3.34 | cytochrome-c oxidase activity | 36 |
| GO:0003724 | 3.15 | RNA helicase activity | 25 |
| GO:0004563 | 2.91 | beta-N-acetylhexosaminidase activity | 34 |
| GO:0030337 | 2.87 | DNA polymerase processivity factor activity | 25 |
| GO:0006275 | 2.87 | regulation of DNA replication | 25 |
| GO:0043626 | 2.87 | PCNA complex | 25 |
| GO:0006629 | 2.82 | lipid metabolic process | 1739 |
| GO:0004784 | 2.79 | superoxide dismutase activity | 20 |
| GO:0004386 | 2.62 | helicase activity | 808 |
| GO:0016884 | 2.62 | carbon-nitrogen ligase activity, with glutamine as amido-N-donor | 230 |
| GO:0016763 | 2.51 | transferase activity, transferring pentosyl groups | 34 |
| GO:0016760 | 2.44 | cellulose synthase (UDP-forming) activity | 321 |
| GO:0030244 | 2.44 | cellulose biosynthetic process | 324 |
| GO:0000902 | 2.44 | cell morphogenesis | 148 |
| GO:0006184 | 2.44 | obsolete GTP catabolic process | 43 |
| GO:0006814 | 2.40 | sodium ion transport | 112 |
| GO:0015105 | 2.39 | arsenite transmembrane transporter activity | 55 |
| GO:0045132 | 2.30 | meiotic chromosome segregation | 34 |
| GO:0012511 | 2.30 | monolayer-surrounded lipid storage body | 35 |
| GO:0004427 | 2.29 | inorganic diphosphatase activity | 50 |
| GO:0016829 | 2.23 | lyase activity | 343 |
| GO:0051087 | 2.18 | chaperone binding | 135 |
| GO:0005739 | 2.18 | mitochondrion | 24 |
| GO:0031072 | 2.03 | heat shock protein binding | 518 |
| GO:0003747 | 2.03 | translation release factor activity | 37 |
| GO:0006415 | 2.03 | translational termination | 37 |
