# Supplementary material for: Genomic prediction applied to high-biomass sorghum for bioenergy production
Source: Mol Breed. 2018 Apr 10;38(4):49. doi: 10.1007/s11032-018-0802-5 (PMC5893689; doi:10.1007/s11032-018-0802-5)
Supplement: Supplementary file 13 — (DOCX 23 kb) [file 11032_2018_802_MOESM13_ESM.docx]

**Online Resource 13**

**Article Title:** Genomic prediction applied to high biomass sorghum for bioenergy production

**Journal:** Molecular Breeding

**Authors:** Amanda Avelar de Oliveira; Maria Marta Pastina; Vander Filipe de Souza; Rafael Augusto da Costa Parrella; Roberto Willians Noda; Maria Lúcia Ferreira Simeone; Robert Eugene Schaffert; Jurandir Vieira de Magalhães; Cynthia Maria Borges Damasceno; Gabriel Rodrigues Alves Margarido.

**Name, affiliation, and email of corresponding author:**

Gabriel Rodrigues Alves Margarido

Escola Superior de Agricultura Luiz de Queiroz, USP

Piracicaba, SP 13418-900, Brazil

e-mail: gramarga@usp.br

Cynthia Maria Borges Damasceno

Embrapa Milho e Sorgo

Sete Lagoas, MG 35701-970, Brazil

e-mail: [cynthia.damasceno@embrapa.br](mailto:cynthia.damasceno@embrapa.br)

**Supplementary Table 16** Results of the functional enrichment Kolmogorov-Smirnov test for the trait hemicellulose. The false discovery rate corrected $p$-value and description for each enriched gene ontology term are shown.

| **GO term** | **- log_10_ p-value** | **Description** | **Number of markers** |
| --- | --- | --- | --- |
| GO:0031227 | 13.09954848 | intrinsic component of endoplasmic reticulum membrane | 91 |
| GO:0006505 | 11.65354445 | GPI anchor metabolic process | 61 |
| GO:0016772 | 10.89968324 | transferase activity, transferring phosphorus-containing groups | 198 |
| GO:0008759 | 10.37203047 | UDP-3-O-[3-hydroxymyristoyl] N-acetylglucosamine deacetylase activity | 16 |
| GO:0009245 | 9.36664179 | lipid A biosynthetic process | 26 |
| GO:0043043 | 8.713494663 | peptide biosynthetic process | 26 |
| GO:0008508 | 8.553861669 | bile acid:sodium symporter activity | 48 |
| GO:0035556 | 8.259763086 | intracellular signal transduction | 120 |
| GO:0000166 | 7.371823172 | nucleotide binding | 1084 |
| GO:0019748 | 7.371823172 | secondary metabolic process | 18 |
| GO:0016884 | 7.31050934 | carbon-nitrogen ligase activity, with glutamine as amido-N-donor | 230 |
| GO:0003746 | 7.117925809 | translation elongation factor activity | 44 |
| GO:0016310 | 6.785071329 | phosphorylation | 100 |
| GO:0055085 | 5.86931793 | transmembrane transport | 4185 |
| GO:0035299 | 5.794339907 | inositol pentakisphosphate 2-kinase activity | 12 |
| GO:0006414 | 5.64013082 | translational elongation | 61 |
| GO:0005975 | 5.615957585 | carbohydrate metabolic process | 2696 |
| GO:0030515 | 5.121934519 | snoRNA binding | 16 |
| GO:0031120 | 5.121934519 | snRNA pseudouridine synthesis | 16 |
| GO:0008652 | 4.9365561 | cellular amino acid biosynthetic process | 154 |
| GO:0004812 | 4.928983166 | aminoacyl-tRNA ligase activity | 320 |
| GO:0005249 | 4.928983166 | voltage-gated potassium channel activity | 31 |
| GO:0008076 | 4.928983166 | voltage-gated potassium channel complex | 31 |
| GO:0004146 | 4.928983166 | dihydrofolate reductase activity | 14 |
| GO:0006545 | 4.928983166 | glycine biosynthetic process | 14 |
| GO:0009165 | 4.928983166 | nucleotide biosynthetic process | 14 |
| GO:0004799 | 4.928983166 | thymidylate synthase activity | 14 |
| GO:0006231 | 4.928983166 | dTMP biosynthetic process | 14 |
| GO:0004345 | 4.806535435 | glucose-6-phosphate dehydrogenase activity | 47 |
| GO:0006006 | 4.806535435 | glucose metabolic process | 47 |
| GO:0003843 | 4.653533423 | 1,3-beta-D-glucan synthase activity | 108 |
| GO:0006075 | 4.653533423 | (1->3)-beta-D-glucan biosynthetic process | 108 |
| GO:0000148 | 4.653533423 | 1,3-beta-D-glucan synthase complex | 108 |
| GO:0015105 | 4.646800196 | arsenite transmembrane transporter activity | 55 |
| GO:0015991 | 4.458248909 | ATP hydrolysis coupled proton transport | 109 |
| GO:0004553 | 4.345488434 | hydrolase activity, hydrolyzing O-glycosyl compounds | 1849 |
| GO:0006520 | 4.330277881 | cellular amino acid metabolic process | 228 |
| GO:0006418 | 4.304511371 | tRNA aminoacylation for protein translation | 296 |
| GO:0042545 | 4.036365848 | cell wall modification | 246 |
| GO:0015137 | 4.030824274 | citrate transmembrane transporter activity | 107 |
| GO:0015746 | 4.030824274 | citrate transport | 107 |
| GO:0030599 | 3.94816608 | pectinesterase activity | 404 |
| GO:0016758 | 3.872728931 | transferase activity, transferring hexosyl groups | 1488 |
| GO:0046961 | 3.843973515 | proton-transporting ATPase activity, rotational mechanism | 91 |
| GO:0004857 | 3.758946967 | enzyme inhibitor activity | 300 |
| GO:0005618 | 3.758727714 | cell wall | 449 |
| GO:0003993 | 3.625576352 | acid phosphatase activity | 92 |
| GO:0050790 | 3.620674067 | regulation of catalytic activity | 9 |
| GO:0008963 | 3.620674067 | phospho-N-acetylmuramoyl-pentapeptide-transferase activity | 18 |
| GO:0005789 | 3.603217512 | endoplasmic reticulum membrane | 53 |
| GO:0003887 | 3.603217512 | DNA-directed DNA polymerase activity | 202 |
| GO:0033178 | 3.463735788 | proton-transporting two-sector ATPase complex, catalytic domain | 75 |
| GO:0045454 | 3.27363176 | cell redox homeostasis | 507 |
| GO:0016810 | 3.22794181 | hydrolase activity, acting on carbon-nitrogen (but not peptide) bonds | 69 |
| GO:0015035 | 3.225354521 | protein disulfide oxidoreductase activity | 249 |
| GO:0008324 | 3.207526712 | cation transmembrane transporter activity | 89 |
| GO:0009029 | 3.123248414 | tetraacyldisaccharide 4'-kinase activity | 8 |
| GO:0003871 | 3.035352533 | 5-methyltetrahydropteroyltriglutamate-homocysteine S-methyltransferase activity | 55 |
| GO:0009086 | 3.035352533 | methionine biosynthetic process | 55 |
| GO:0007264 | 3.012240259 | small GTPase mediated signal transduction | 329 |
| GO:0009439 | 2.997284147 | cyanate metabolic process | 8 |
| GO:0007165 | 2.996521157 | signal transduction | 473 |
| GO:0015992 | 2.992707472 | proton transport | 49 |
| GO:0008408 | 2.936309946 | 3'-5' exonuclease activity | 102 |
| GO:0004565 | 2.901199814 | beta-galactosidase activity | 92 |
| GO:0009341 | 2.901199814 | beta-galactosidase complex | 92 |
| GO:0005509 | 2.727551876 | calcium ion binding | 673 |
| GO:0015923 | 2.702043703 | mannosidase activity | 67 |
| GO:0006013 | 2.702043703 | mannose metabolic process | 67 |
| GO:0005856 | 2.702043703 | cytoskeleton | 68 |
| GO:0015078 | 2.656651243 | hydrogen ion transmembrane transporter activity | 30 |
| GO:0004143 | 2.620144554 | diacylglycerol kinase activity | 88 |
| GO:0007205 | 2.620144554 | protein kinase C-activating G-protein coupled receptor signaling pathway | 88 |
| GO:0005515 | 2.606443466 | protein binding | 14625 |
| GO:0004650 | 2.597067451 | polygalacturonase activity | 318 |
| GO:0050661 | 2.597067451 | NADP binding | 230 |
| GO:0004066 | 2.52982506 | asparagine synthase (glutamine-hydrolyzing) activity | 82 |
| GO:0006529 | 2.52982506 | asparagine biosynthetic process | 82 |
| GO:0016567 | 2.50386434 | protein ubiquitination | 469 |
| GO:0000151 | 2.50386434 | ubiquitin ligase complex | 469 |
| GO:0005351 | 2.50386434 | sugar:proton symporter activity | 52 |
| GO:0008643 | 2.50386434 | carbohydrate transport | 52 |
| GO:0009378 | 2.483824525 | four-way junction helicase activity | 232 |
| GO:0015238 | 2.448228754 | drug transmembrane transporter activity | 492 |
| GO:0015297 | 2.448228754 | antiporter activity | 492 |
| GO:0006855 | 2.448228754 | drug transmembrane transport | 492 |
| GO:0019001 | 2.448228754 | guanyl nucleotide binding | 64 |
| GO:0007186 | 2.448228754 | G-protein coupled receptor signaling pathway | 64 |
| GO:0006506 | 2.448228754 | GPI anchor biosynthetic process | 59 |
| GO:0008131 | 2.446679746 | primary amine oxidase activity | 24 |
| GO:0009308 | 2.446679746 | amine metabolic process | 24 |
| GO:0006450 | 2.414265094 | regulation of translational fidelity | 11 |
| GO:0004869 | 2.414265094 | cysteine-type endopeptidase inhibitor activity | 35 |
| GO:0000139 | 2.404513929 | Golgi membrane | 61 |
| GO:0016300 | 2.376776469 | tRNA (uracil) methyltransferase activity | 24 |
| GO:0002098 | 2.376776469 | tRNA wobble uridine modification | 24 |
| GO:0006281 | 2.359733499 | DNA repair | 634 |
| GO:0016070 | 2.347419229 | RNA metabolic process | 50 |
| GO:0008374 | 2.325859969 | O-acyltransferase activity | 94 |
| GO:0004559 | 2.30195963 | alpha-mannosidase activity | 70 |
| GO:0045300 | 2.234637904 | acyl-[acyl-carrier-protein] desaturase activity | 42 |
| GO:0004540 | 2.221691117 | ribonuclease activity | 57 |
| GO:0016740 | 2.218896418 | transferase activity | 386 |
| GO:0042254 | 2.205442747 | ribosome biogenesis | 63 |
| GO:0005759 | 2.197600307 | mitochondrial matrix | 21 |
| GO:0004842 | 2.193530395 | ubiquitin-protein transferase activity | 481 |
| GO:0016773 | 2.166078754 | phosphotransferase activity, alcohol group as acceptor | 523 |
| GO:0030833 | 2.162633075 | regulation of actin filament polymerization | 44 |
| GO:0004176 | 2.156867149 | ATP-dependent peptidase activity | 103 |
| GO:0005525 | 2.149774395 | GTP binding | 919 |
| GO:0006909 | 2.088309637 | phagocytosis | 21 |
| GO:0006310 | 2.035943502 | DNA recombination | 274 |
| GO:0004190 | 2.020707192 | aspartic-type endopeptidase activity | 821 |
| GO:0008152 | 2.006712677 | metabolic process | 6804 |
| GO:0046034 | 2.006712677 | ATP metabolic process | 27 |
| GO:0016469 | 2.006712677 | proton-transporting two-sector ATPase complex | 27 |
