# Supplementary material for: Genomic prediction applied to high-biomass sorghum for bioenergy production
Source: Mol Breed. 2018 Apr 10;38(4):49. doi: 10.1007/s11032-018-0802-5 (PMC5893689; doi:10.1007/s11032-018-0802-5)
Supplement: Supplementary file 14 — (DOCX 19 kb) [file 11032_2018_802_MOESM14_ESM.docx]

**Online Resource 14**

**Article Title:** Genomic prediction applied to high biomass sorghum for bioenergy production

**Journal:** Molecular Breeding

**Authors:** Amanda Avelar de Oliveira; Maria Marta Pastina; Vander Filipe de Souza; Rafael Augusto da Costa Parrella; Roberto Willians Noda; Maria Lúcia Ferreira Simeone; Robert Eugene Schaffert; Jurandir Vieira de Magalhães; Cynthia Maria Borges Damasceno; Gabriel Rodrigues Alves Margarido.

**Name, affiliation, and email of corresponding author:**

Gabriel Rodrigues Alves Margarido

Escola Superior de Agricultura Luiz de Queiroz, USP

Piracicaba, SP 13418-900, Brazil

e-mail: gramarga@usp.br

Cynthia Maria Borges Damasceno

Embrapa Milho e Sorgo

Sete Lagoas, MG 35701-970, Brazil

e-mail: [cynthia.damasceno@embrapa.br](mailto:cynthia.damasceno@embrapa.br)

**Supplementary Table 17** Results of the functional enrichment Kolmogorov-Smirnov test for the trait lignin. The false discovery rate corrected $p$-value and description for each enriched gene ontology term are shown.

| **GO term** | **- log_10_ p-value** | **Description** | **Number of markers** |
| --- | --- | --- | --- |
| GO:0043169 | 9.22 | cation binding | 127 |
| GO:0000042 | 7.73 | protein targeting to Golgi | 18 |
| GO:0004176 | 7.64 | ATP-dependent peptidase activity | 103 |
| GO:0003824 | 7.23 | catalytic activity | 4286 |
| GO:0006914 | 7.23 | autophagy | 101 |
| GO:0000166 | 6.90 | nucleotide binding | 1084 |
| GO:0008759 | 6.90 | UDP-3-O-[3-hydroxymyristoyl] N-acetylglucosamine deacetylase activity | 16 |
| GO:0046872 | 6.68 | metal ion binding | 1649 |
| GO:0031227 | 6.45 | intrinsic component of endoplasmic reticulum membrane | 91 |
| GO:0003937 | 6.07 | IMP cyclohydrolase activity | 36 |
| GO:0004643 | 6.07 | phosphoribosylaminoimidazolecarboxamide formyltransferase activity | 36 |
| GO:0006505 | 6.05 | GPI anchor metabolic process | 61 |
| GO:0006164 | 5.68 | purine nucleotide biosynthetic process | 41 |
| GO:0008104 | 5.31 | protein localization | 94 |
| GO:0000145 | 4.99 | exocyst | 243 |
| GO:0035556 | 4.99 | intracellular signal transduction | 120 |
| GO:0005351 | 4.99 | sugar:proton symporter activity | 52 |
| GO:0008643 | 4.99 | carbohydrate transport | 52 |
| GO:0003723 | 4.72 | RNA binding | 1179 |
| GO:0006418 | 4.49 | tRNA aminoacylation for protein translation | 296 |
| GO:0009245 | 4.49 | lipid A biosynthetic process | 26 |
| GO:0004812 | 4.49 | aminoacyl-tRNA ligase activity | 320 |
| GO:0006887 | 4.12 | exocytosis | 245 |
| GO:0004325 | 3.97 | ferrochelatase activity | 15 |
| GO:0006783 | 3.97 | heme biosynthetic process | 15 |
| GO:0006508 | 3.95 | proteolysis | 2781 |
| GO:0015105 | 3.88 | arsenite transmembrane transporter activity | 55 |
| GO:0008237 | 3.79 | metallopeptidase activity | 73 |
| GO:0004096 | 3.75 | catalase activity | 67 |
| GO:0019370 | 3.59 | leukotriene biosynthetic process | 12 |
| GO:0030259 | 3.59 | lipid glycosylation | 52 |
| GO:0051082 | 3.42 | unfolded protein binding | 166 |
| GO:0000774 | 3.41 | adenyl-nucleotide exchange factor activity | 41 |
| GO:0042803 | 3.41 | protein homodimerization activity | 41 |
| GO:0000139 | 3.35 | Golgi membrane | 61 |
| GO:0016998 | 3.28 | cell wall macromolecule catabolic process | 176 |
| GO:0015137 | 3.28 | citrate transmembrane transporter activity | 107 |
| GO:0015746 | 3.28 | citrate transport | 107 |
| GO:0004652 | 3.21 | polynucleotide adenylyltransferase activity | 59 |
| GO:0043631 | 3.21 | RNA polyadenylation | 59 |
| GO:0006694 | 3.09 | steroid biosynthetic process | 716 |
| GO:0003849 | 2.93 | 3-deoxy-7-phosphoheptulonate synthase activity | 24 |
| GO:0006813 | 2.87 | potassium ion transport | 53 |
| GO:0008508 | 2.86 | bile acid:sodium symporter activity | 48 |
| GO:0019538 | 2.82 | protein metabolic process | 108 |
| GO:0048544 | 2.82 | recognition of pollen | 531 |
| GO:0006351 | 2.81 | transcription, DNA-templated | 520 |
| GO:0008375 | 2.71 | acetylglucosaminyltransferase activity | 239 |
| GO:0008963 | 2.66 | phospho-N-acetylmuramoyl-pentapeptide-transferase activity | 18 |
| GO:0005975 | 2.64 | carbohydrate metabolic process | 2696 |
| GO:0010277 | 2.64 | chlorophyllide a oxygenase [overall] activity | 38 |
| GO:0004348 | 2.62 | glucosylceramidase activity | 11 |
| GO:0006665 | 2.62 | sphingolipid metabolic process | 11 |
| GO:0005672 | 2.60 | transcription factor TFIIA complex | 14 |
| GO:0006260 | 2.57 | DNA replication | 284 |
| GO:0004519 | 2.50 | endonuclease activity | 75 |
| GO:0015923 | 2.45 | mannosidase activity | 67 |
| GO:0006013 | 2.45 | mannose metabolic process | 67 |
| GO:0004146 | 2.45 | dihydrofolate reductase activity | 14 |
| GO:0006545 | 2.45 | glycine biosynthetic process | 14 |
| GO:0009165 | 2.45 | nucleotide biosynthetic process | 14 |
| GO:0004799 | 2.45 | thymidylate synthase activity | 14 |
| GO:0006231 | 2.45 | dTMP biosynthetic process | 14 |
| GO:0004185 | 2.34 | serine-type carboxypeptidase activity | 381 |
| GO:0006812 | 2.33 | cation transport | 263 |
| GO:0003854 | 2.30 | 3-beta-hydroxy-delta5-steroid dehydrogenase activity | 675 |
| GO:0016616 | 2.22 | oxidoreductase activity, acting on the CH-OH group of donors, NAD or NADP as acceptor | 990 |
| GO:0009435 | 2.20 | NAD biosynthetic process | 37 |
| GO:0004435 | 2.18 | phosphatidylinositol phospholipase C activity | 31 |
| GO:0009073 | 2.17 | aromatic amino acid family biosynthetic process | 29 |
| GO:0004559 | 2.15 | alpha-mannosidase activity | 70 |
| GO:0004518 | 2.13 | nuclease activity | 107 |
| GO:0055085 | 2.10 | transmembrane transport | 4185 |
| GO:0006457 | 2.08 | protein folding | 525 |
| GO:0016844 | 2.08 | strictosidine synthase activity | 105 |
| GO:0004655 | 2.05 | porphobilinogen synthase activity | 25 |
| GO:0005247 | 2.01 | voltage-gated chloride channel activity | 95 |
| GO:0006821 | 2.01 | chloride transport | 95 |
