# Supplementary material for: Genomic prediction applied to high-biomass sorghum for bioenergy production
Source: Mol Breed. 2018 Apr 10;38(4):49. doi: 10.1007/s11032-018-0802-5 (PMC5893689; doi:10.1007/s11032-018-0802-5)
Supplement: Supplementary file 15 — (DOCX 21 kb) [file 11032_2018_802_MOESM15_ESM.docx]

**Online Resource 15**

**Article Title:** Genomic prediction applied to high biomass sorghum for bioenergy production

**Journal:** Molecular Breeding

**Authors:** Amanda Avelar de Oliveira; Maria Marta Pastina; Vander Filipe de Souza; Rafael Augusto da Costa Parrella; Roberto Willians Noda; Maria Lúcia Ferreira Simeone; Robert Eugene Schaffert; Jurandir Vieira de Magalhães; Cynthia Maria Borges Damasceno; Gabriel Rodrigues Alves Margarido.

**Name, affiliation, and email of corresponding author:**

Gabriel Rodrigues Alves Margarido

Escola Superior de Agricultura Luiz de Queiroz, USP

Piracicaba, SP 13418-900, Brazil

e-mail: gramarga@usp.br

Cynthia Maria Borges Damasceno

Embrapa Milho e Sorgo

Sete Lagoas, MG 35701-970, Brazil

e-mail: [cynthia.damasceno@embrapa.br](mailto:cynthia.damasceno@embrapa.br)

**Supplementary Table 18** Results of the functional enrichment Kolmogorov-Smirnov test for the trait dry matter yield. The false discovery rate corrected $p$-value and description for each enriched gene ontology term are shown.

| **GO term** | **-log_10_ p-value** | **Description** | **Number of markers** |
| --- | --- | --- | --- |
| GO:0009055 | 11.30 | electron carrier activity | 3340 |
| GO:0005506 | 9.82 | iron ion binding | 2567 |
| GO:0005351 | 9.48 | sugar:proton symporter activity | 52 |
| GO:0008643 | 9.48 | carbohydrate transport | 52 |
| GO:0000139 | 8.73 | Golgi membrane | 61 |
| GO:0006807 | 8.49 | nitrogen compound metabolic process | 111 |
| GO:0004146 | 7.92 | dihydrofolate reductase activity | 14 |
| GO:0006545 | 7.92 | glycine biosynthetic process | 14 |
| GO:0009165 | 7.92 | nucleotide biosynthetic process | 14 |
| GO:0004799 | 7.92 | thymidylate synthase activity | 14 |
| GO:0006231 | 7.92 | dTMP biosynthetic process | 14 |
| GO:0008759 | 7.87 | UDP-3-O-[3-hydroxymyristoyl] N-acetylglucosamine deacetylase activity | 16 |
| GO:0015035 | 7.65 | protein disulfide oxidoreductase activity | 249 |
| GO:0016705 | 7.09 | oxidoreductase activity, acting on paired donors, with incorporation or reduction of molecular oxygen | 2242 |
| GO:0020037 | 7.02 | heme binding | 3066 |
| GO:0009245 | 6.58 | lipid A biosynthetic process | 26 |
| GO:0008104 | 6.30 | protein localization | 94 |
| GO:0045454 | 6.16 | cell redox homeostasis | 507 |
| GO:0005622 | 5.77 | intracellular | 2375 |
| GO:0008652 | 5.77 | cellular amino acid biosynthetic process | 154 |
| GO:0016810 | 5.73 | hydrolase activity, acting on carbon-nitrogen (but not peptide) bonds | 69 |
| GO:0005086 | 5.49 | ARF guanyl-nucleotide exchange factor activity | 38 |
| GO:0032012 | 5.49 | regulation of ARF protein signal transduction | 38 |
| GO:0003724 | 5.33 | RNA helicase activity | 25 |
| GO:0019748 | 5.25 | secondary metabolic process | 18 |
| GO:0016310 | 4.80 | phosphorylation | 100 |
| GO:0055114 | 4.78 | oxidation-reduction process | 8245 |
| GO:0043531 | 4.78 | ADP binding | 2507 |
| GO:0006810 | 4.78 | transport | 1534 |
| GO:0008601 | 4.78 | protein phosphatase type 2A regulator activity | 63 |
| GO:0000159 | 4.78 | protein phosphatase type 2A complex | 63 |
| GO:0009982 | 4.70 | pseudouridine synthase activity | 95 |
| GO:0001522 | 4.70 | pseudouridine synthesis | 95 |
| GO:0017176 | 4.52 | phosphatidylinositol N-acetylglucosaminyltransferase activity | 24 |
| GO:0006506 | 4.40 | GPI anchor biosynthetic process | 59 |
| GO:0003993 | 4.23 | acid phosphatase activity | 92 |
| GO:0004540 | 4.19 | ribonuclease activity | 57 |
| GO:0012511 | 3.98 | monolayer-surrounded lipid storage body | 35 |
| GO:0004525 | 3.91 | ribonuclease III activity | 215 |
| GO:0016772 | 3.87 | transferase activity, transferring phosphorus-containing groups | 198 |
| GO:0035556 | 3.81 | intracellular signal transduction | 120 |
| GO:0006450 | 3.80 | regulation of translational fidelity | 11 |
| GO:0008234 | 3.54 | cysteine-type peptidase activity | 391 |
| GO:0005801 | 3.52 | cis-Golgi network | 27 |
| GO:0016021 | 3.51 | integral component of membrane | 4252 |
| GO:0004806 | 3.44 | triglyceride lipase activity | 379 |
| GO:0016884 | 3.37 | carbon-nitrogen ligase activity, with glutamine as amido-N-donor | 230 |
| GO:0016616 | 3.31 | oxidoreductase activity, acting on the CH-OH group of donors, NAD or NADP as acceptor | 990 |
| GO:0005215 | 3.25 | transporter activity | 1529 |
| GO:0009451 | 3.18 | RNA modification | 112 |
| GO:0008081 | 3.18 | phosphoric diester hydrolase activity | 85 |
| GO:0006633 | 3.14 | fatty acid biosynthetic process | 633 |
| GO:0006468 | 3.12 | protein phosphorylation | 9161 |
| GO:0004672 | 3.00 | protein kinase activity | 9137 |
| GO:0019139 | 2.94 | cytokinin dehydrogenase activity | 57 |
| GO:0009690 | 2.94 | cytokinin metabolic process | 57 |
| GO:0006383 | 2.94 | transcription from RNA polymerase III promoter | 8 |
| GO:0005666 | 2.94 | DNA-directed RNA polymerase III complex | 8 |
| GO:0004559 | 2.93 | alpha-mannosidase activity | 70 |
| GO:0008380 | 2.88 | RNA splicing | 10 |
| GO:0016746 | 2.78 | transferase activity, transferring acyl groups | 615 |
| GO:0007275 | 2.78 | multicellular organismal development | 277 |
| GO:0015923 | 2.78 | mannosidase activity | 67 |
| GO:0006013 | 2.78 | mannose metabolic process | 67 |
| GO:0005524 | 2.75 | ATP binding | 14385 |
| GO:0006511 | 2.66 | ubiquitin-dependent protein catabolic process | 509 |
| GO:0003871 | 2.64 | 5-methyltetrahydropteroyltriglutamate-homocysteine S-methyltransferase activity | 55 |
| GO:0009086 | 2.64 | methionine biosynthetic process | 55 |
| GO:0008963 | 2.64 | phospho-N-acetylmuramoyl-pentapeptide-transferase activity | 18 |
| GO:0033615 | 2.64 | mitochondrial proton-transporting ATP synthase complex assembly | 20 |
| GO:0016740 | 2.55 | transferase activity | 386 |
| GO:0016872 | 2.52 | intramolecular lyase activity | 22 |
| GO:0042398 | 2.52 | cellular modified amino acid biosynthetic process | 22 |
| GO:0005337 | 2.48 | nucleoside transmembrane transporter activity | 19 |
| GO:0003913 | 2.48 | DNA photolyase activity | 63 |
| GO:0003937 | 2.48 | IMP cyclohydrolase activity | 36 |
| GO:0004643 | 2.48 | phosphoribosylaminoimidazolecarboxamide formyltransferase activity | 36 |
| GO:0044267 | 2.45 | cellular protein metabolic process | 118 |
| GO:0004514 | 2.44 | nicotinate-nucleotide diphosphorylase (carboxylating) activity | 10 |
| GO:0044237 | 2.34 | cellular metabolic process | 922 |
| GO:0006164 | 2.34 | purine nucleotide biosynthetic process | 41 |
| GO:0007264 | 2.33 | small GTPase mediated signal transduction | 329 |
| GO:0043169 | 2.31 | cation binding | 127 |
| GO:0004834 | 2.28 | tryptophan synthase activity | 13 |
| GO:0008837 | 2.27 | diaminopimelate epimerase activity | 6 |
| GO:0050662 | 2.25 | coenzyme binding | 1041 |
| GO:0004003 | 2.24 | ATP-dependent DNA helicase activity | 56 |
| GO:0004096 | 2.23 | catalase activity | 67 |
| GO:0005543 | 2.15 | phospholipid binding | 186 |
| GO:0004832 | 2.15 | valine-tRNA ligase activity | 12 |
| GO:0006438 | 2.15 | valyl-tRNA aminoacylation | 12 |
| GO:0003755 | 2.13 | peptidyl-prolyl cis-trans isomerase activity | 149 |
| GO:0005739 | 2.12 | mitochondrion | 24 |
| GO:2001070 | 2.11 | starch binding | 73 |
| GO:0003725 | 2.10 | double-stranded RNA binding | 265 |
| GO:0030337 | 2.02 | DNA polymerase processivity factor activity | 25 |
| GO:0006275 | 2.02 | regulation of DNA replication | 25 |
| GO:0043626 | 2.02 | PCNA complex | 25 |
| GO:0031072 | 2.01 | heat shock protein binding | 518 |
| GO:0051287 | 2.01 | NAD binding | 216 |
| GO:0006520 | 2.01 | cellular amino acid metabolic process | 228 |
