# Supplementary material for: Genomic prediction applied to high-biomass sorghum for bioenergy production
Source: Mol Breed. 2018 Apr 10;38(4):49. doi: 10.1007/s11032-018-0802-5 (PMC5893689; doi:10.1007/s11032-018-0802-5)
Supplement: Supplementary file 16 — (DOCX 22 kb) [file 11032_2018_802_MOESM16_ESM.docx]

**Online Resource 16**

**Article Title:** Genomic prediction applied to high biomass sorghum for bioenergy production

**Journal:** Molecular Breeding

**Authors:** Amanda Avelar de Oliveira; Maria Marta Pastina; Vander Filipe de Souza; Rafael Augusto da Costa Parrella; Roberto Willians Noda; Maria Lúcia Ferreira Simeone; Robert Eugene Schaffert; Jurandir Vieira de Magalhães; Cynthia Maria Borges Damasceno; Gabriel Rodrigues Alves Margarido.

**Name, affiliation, and email of corresponding author:**

Gabriel Rodrigues Alves Margarido

Escola Superior de Agricultura Luiz de Queiroz, USP

Piracicaba, SP 13418-900, Brazil

e-mail: gramarga@usp.br

Cynthia Maria Borges Damasceno

Embrapa Milho e Sorgo

Sete Lagoas, MG 35701-970, Brazil

e-mail: [cynthia.damasceno@embrapa.br](mailto:cynthia.damasceno@embrapa.br)

**Supplementary Table 19** Results of the functional enrichment Kolmogorov-Smirnov test for the trait fresh matter yield. The false discovery rate corrected $p$-value and description for each enriched gene ontology term are shown.

| **GO term** | **- log_10_ p-value** | **Description** | **Number of markers** |
| --- | --- | --- | --- |
| GO:0016616 | 8.12 | oxidoreductase activity, acting on the CH-OH group of donors, NAD or NADP as acceptor | 990 |
| GO:0004146 | 7.73 | dihydrofolate reductase activity | 14 |
| GO:0006545 | 7.73 | glycine biosynthetic process | 14 |
| GO:0009165 | 7.73 | nucleotide biosynthetic process | 14 |
| GO:0004799 | 7.73 | thymidylate synthase activity | 14 |
| GO:0006231 | 7.73 | dTMP biosynthetic process | 14 |
| GO:0019748 | 7.05 | secondary metabolic process | 18 |
| GO:0005215 | 6.99 | transporter activity | 1529 |
| GO:0003724 | 6.45 | RNA helicase activity | 25 |
| GO:0006468 | 5.97 | protein phosphorylation | 9161 |
| GO:0044267 | 5.97 | cellular protein metabolic process | 118 |
| GO:0004672 | 5.78 | protein kinase activity | 9137 |
| GO:0016310 | 5.69 | phosphorylation | 100 |
| GO:0008759 | 5.34 | UDP-3-O-[3-hydroxymyristoyl] N-acetylglucosamine deacetylase activity | 16 |
| GO:0005351 | 5.22 | sugar:proton symporter activity | 52 |
| GO:0008643 | 5.22 | carbohydrate transport | 52 |
| GO:0035299 | 5.22 | inositol pentakisphosphate 2-kinase activity | 12 |
| GO:0000139 | 5.16 | Golgi membrane | 61 |
| GO:0009451 | 5.00 | RNA modification | 112 |
| GO:0005506 | 4.93 | iron ion binding | 2567 |
| GO:0009245 | 4.56 | lipid A biosynthetic process | 26 |
| GO:0006450 | 4.52 | regulation of translational fidelity | 11 |
| GO:0003899 | 4.49 | DNA-directed RNA polymerase activity | 325 |
| GO:0009982 | 4.49 | pseudouridine synthase activity | 95 |
| GO:0001522 | 4.49 | pseudouridine synthesis | 95 |
| GO:0055114 | 4.49 | oxidation-reduction process | 8245 |
| GO:0004525 | 3.91 | ribonuclease III activity | 215 |
| GO:0017176 | 3.89 | phosphatidylinositol N-acetylglucosaminyltransferase activity | 24 |
| GO:0004834 | 3.87 | tryptophan synthase activity | 13 |
| GO:0009055 | 3.71 | electron carrier activity | 3340 |
| GO:0012511 | 3.71 | monolayer-surrounded lipid storage body | 35 |
| GO:0006857 | 3.69 | oligopeptide transport | 814 |
| GO:0008081 | 3.63 | phosphoric diester hydrolase activity | 85 |
| GO:0043531 | 3.43 | ADP binding | 2507 |
| GO:0016772 | 3.43 | transferase activity, transferring phosphorus-containing groups | 198 |
| GO:0005622 | 3.42 | intracellular | 2375 |
| GO:0006568 | 3.41 | tryptophan metabolic process | 14 |
| GO:0003854 | 3.39 | 3-beta-hydroxy-delta5-steroid dehydrogenase activity | 675 |
| GO:0005086 | 3.39 | ARF guanyl-nucleotide exchange factor activity | 38 |
| GO:0032012 | 3.39 | regulation of ARF protein signal transduction | 38 |
| GO:0007264 | 3.34 | small GTPase mediated signal transduction | 329 |
| GO:0046961 | 3.21 | proton-transporting ATPase activity, rotational mechanism | 91 |
| GO:0016705 | 3.12 | oxidoreductase activity, acting on paired donors, with incorporation or reduction of molecular oxygen | 2242 |
| GO:0005198 | 3.12 | structural molecule activity | 197 |
| GO:0045454 | 3.07 | cell redox homeostasis | 507 |
| GO:0008652 | 3.07 | cellular amino acid biosynthetic process | 154 |
| GO:0051287 | 3.07 | NAD binding | 216 |
| GO:0016881 | 2.99 | acid-amino acid ligase activity | 266 |
| GO:0006414 | 2.98 | translational elongation | 61 |
| GO:0008963 | 2.98 | phospho-N-acetylmuramoyl-pentapeptide-transferase activity | 18 |
| GO:0033615 | 2.98 | mitochondrial proton-transporting ATP synthase complex assembly | 20 |
| GO:0004553 | 2.96 | hydrolase activity, hydrolyzing O-glycosyl compounds | 1849 |
| GO:0005509 | 2.96 | calcium ion binding | 673 |
| GO:0005739 | 2.96 | mitochondrion | 24 |
| GO:0003725 | 2.89 | double-stranded RNA binding | 265 |
| GO:0050790 | 2.88 | regulation of catalytic activity | 9 |
| GO:0005247 | 2.84 | voltage-gated chloride channel activity | 95 |
| GO:0006821 | 2.84 | chloride transport | 95 |
| GO:0006511 | 2.84 | ubiquitin-dependent protein catabolic process | 509 |
| GO:0015923 | 2.83 | mannosidase activity | 67 |
| GO:0006013 | 2.83 | mannose metabolic process | 67 |
| GO:0015991 | 2.80 | ATP hydrolysis coupled proton transport | 109 |
| GO:0004559 | 2.76 | alpha-mannosidase activity | 70 |
| GO:0006520 | 2.68 | cellular amino acid metabolic process | 228 |
| GO:0006506 | 2.65 | GPI anchor biosynthetic process | 59 |
| GO:0020037 | 2.63 | heme binding | 3066 |
| GO:0016740 | 2.52 | transferase activity | 386 |
| GO:0006694 | 2.52 | steroid biosynthetic process | 716 |
| GO:0005801 | 2.49 | cis-Golgi network | 27 |
| GO:0008104 | 2.48 | protein localization | 94 |
| GO:0006810 | 2.46 | transport | 1534 |
| GO:0005524 | 2.39 | ATP binding | 14385 |
| GO:0009058 | 2.39 | biosynthetic process | 1537 |
| GO:0003993 | 2.38 | acid phosphatase activity | 92 |
| GO:0016021 | 2.37 | integral component of membrane | 4252 |
| GO:0031072 | 2.37 | heat shock protein binding | 518 |
| GO:0016311 | 2.36 | dephosphorylation | 22 |
| GO:0009678 | 2.36 | hydrogen-translocating pyrophosphatase activity | 17 |
| GO:0008380 | 2.34 | RNA splicing | 10 |
| GO:0005337 | 2.34 | nucleoside transmembrane transporter activity | 19 |
| GO:0033178 | 2.34 | proton-transporting two-sector ATPase complex, catalytic domain | 75 |
| GO:0004175 | 2.26 | endopeptidase activity | 24 |
| GO:0019773 | 2.26 | proteasome core complex, alpha-subunit complex | 24 |
| GO:0003916 | 2.26 | DNA topoisomerase activity | 49 |
| GO:0031227 | 2.24 | intrinsic component of endoplasmic reticulum membrane | 91 |
| GO:0015035 | 2.23 | protein disulfide oxidoreductase activity | 249 |
| GO:0008601 | 2.23 | protein phosphatase type 2A regulator activity | 63 |
| GO:0000159 | 2.23 | protein phosphatase type 2A complex | 63 |
| GO:0030515 | 2.15 | snoRNA binding | 16 |
| GO:0031120 | 2.15 | snRNA pseudouridine synthesis | 16 |
| GO:0030288 | 2.13 | outer membrane-bounded periplasmic space | 207 |
| GO:0005798 | 2.13 | Golgi-associated vesicle | 9 |
| GO:0030337 | 2.13 | DNA polymerase processivity factor activity | 25 |
| GO:0006275 | 2.13 | regulation of DNA replication | 25 |
| GO:0043626 | 2.13 | PCNA complex | 25 |
| GO:0003746 | 2.08 | translation elongation factor activity | 44 |
| GO:0005992 | 2.08 | trehalose biosynthetic process | 135 |
| GO:0004970 | 2.08 | ionotropic glutamate receptor activity | 205 |
| GO:0005234 | 2.08 | extracellular-glutamate-gated ion channel activity | 205 |
| GO:0006265 | 2.07 | DNA topological change | 63 |
| GO:0044237 | 2.06 | cellular metabolic process | 922 |
| GO:0019139 | 2.06 | cytokinin dehydrogenase activity | 57 |
| GO:0009690 | 2.06 | cytokinin metabolic process | 57 |
| GO:0004089 | 2.05 | carbonate dehydratase activity | 105 |
| GO:0005694 | 2.01 | chromosome | 74 |
| GO:0043043 | 2.01 | peptide biosynthetic process | 26 |
