# Supplementary material for: Genomic prediction applied to high-biomass sorghum for bioenergy production
Source: Mol Breed. 2018 Apr 10;38(4):49. doi: 10.1007/s11032-018-0802-5 (PMC5893689; doi:10.1007/s11032-018-0802-5)
Supplement: Supplementary file 20 — (DOCX 23 kb) [file 11032_2018_802_MOESM20_ESM.docx]

**Online Resource 20**

**Article Title:** Genomic prediction applied to high biomass sorghum for bioenergy production

**Journal:** Molecular Breeding

**Authors:** Amanda Avelar de Oliveira; Maria Marta Pastina; Vander Filipe de Souza; Rafael Augusto da Costa Parrella; Roberto Willians Noda; Maria Lúcia Ferreira Simeone; Robert Eugene Schaffert; Jurandir Vieira de Magalhães; Cynthia Maria Borges Damasceno; Gabriel Rodrigues Alves Margarido.

**Name, affiliation, and email of corresponding author:**

Gabriel Rodrigues Alves Margarido

Escola Superior de Agricultura Luiz de Queiroz, USP

Piracicaba, SP 13418-900, Brazil

e-mail: gramarga@usp.br

Cynthia Maria Borges Damasceno

Embrapa Milho e Sorgo

Sete Lagoas, MG 35701-970, Brazil

e-mail: [cynthia.damasceno@embrapa.br](mailto:cynthia.damasceno@embrapa.br)


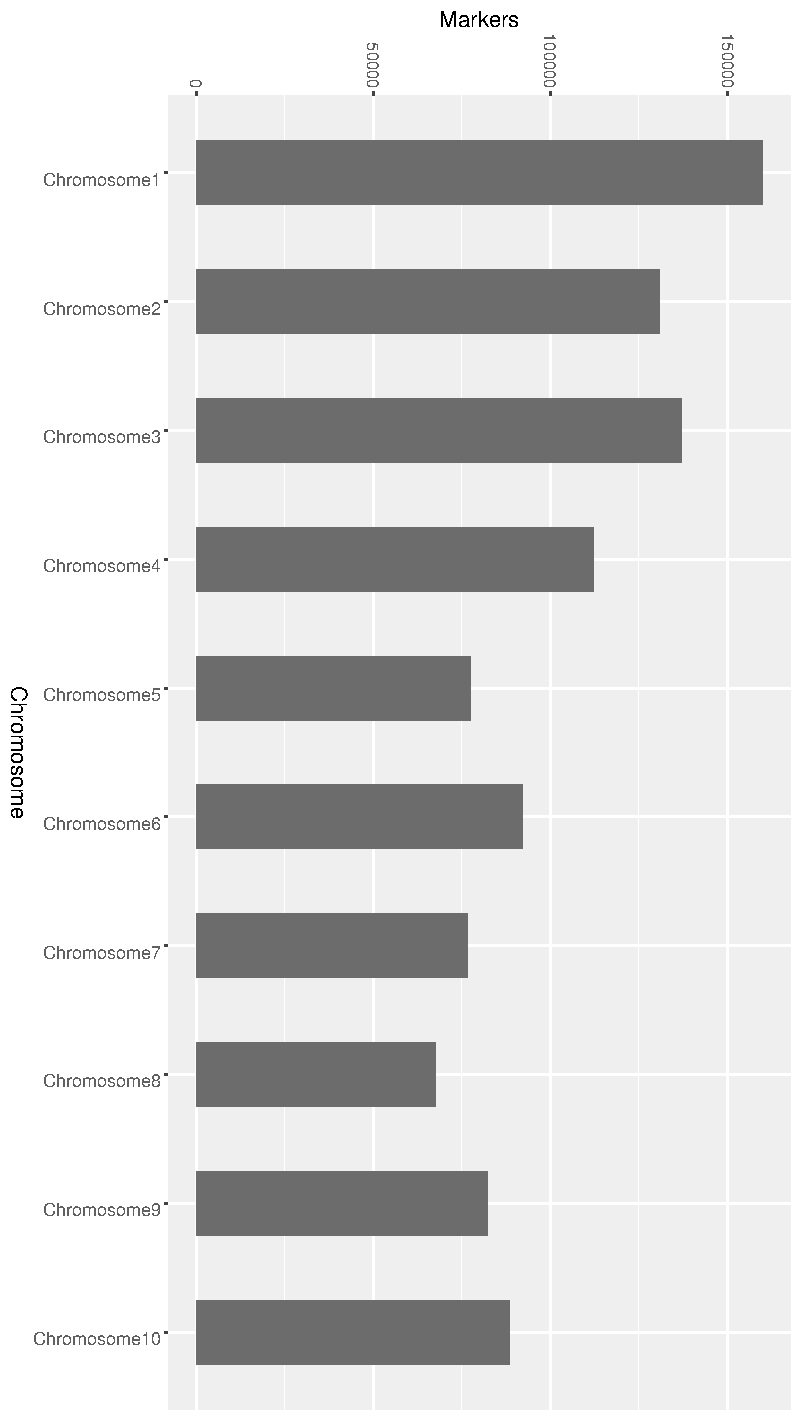


**Supplementary Figure 4** Distribution of 1,024,892 markers by 10 sorghum chromosomes
